# Genetic Predisposition to an Impaired Metabolism of the Branched-Chain Amino Acids and Risk of Type 2 Diabetes: A Mendelian Randomisation Analysis

**DOI:** 10.1371/journal.pmed.1002179

**Published:** 2016-11-29

**Authors:** Luca A. Lotta, Robert A. Scott, Stephen J. Sharp, Stephen Burgess, Jian’an Luan, Therese Tillin, Amand F. Schmidt, Fumiaki Imamura, Isobel D. Stewart, John R. B. Perry, Luke Marney, Albert Koulman, Edward D. Karoly, Nita G. Forouhi, Rasmus J. O. Sjögren, Erik Näslund, Juleen R. Zierath, Anna Krook, David B. Savage, Julian L. Griffin, Nishi Chaturvedi, Aroon D. Hingorani, Kay-Tee Khaw, Inês Barroso, Mark I. McCarthy, Stephen O’Rahilly, Nicholas J. Wareham, Claudia Langenberg

**Affiliations:** 1 MRC Epidemiology Unit, University of Cambridge, Cambridge, United Kingdom; 2 Department of Public Health and Primary Care, University of Cambridge, Cambridge, United Kingdom; 3 Institute of Cardiovascular Science, Faculty of Population Health, University College London, London, United Kingdom; 4 MRC Human Nutrition Research, Cambridge, United Kingdom; 5 Metabolon, Morrisville, North Carolina, United States of America; 6 Department of Molecular Medicine and Surgery, Karolinska Institutet, Stockholm, Sweden; 7 Division of Surgery, Department of Clinical Sciences, Danderyd Hospital, Karolinska Institutet, Stockholm, Sweden; 8 Department of Physiology and Pharmacology, Karolinska Institutet, Stockholm, Sweden; 9 Metabolic Research Laboratories, Institute of Metabolic Science, University of Cambridge, Cambridge, United Kingdom; 10 Department of Biochemistry, University of Cambridge, Cambridge, United Kingdom; 11 Wellcome Trust Sanger Institute, Cambridge, United Kingdom; 12 Oxford Centre for Diabetes, Endocrinology and Metabolism, and Wellcome Trust Centre for Human Genetics, University of Oxford, Oxford, United Kingdom; Imperial College London, UNITED KINGDOM

## Abstract

**Background:**

Higher circulating levels of the branched-chain amino acids (BCAAs; i.e., isoleucine, leucine, and valine) are strongly associated with higher type 2 diabetes risk, but it is not known whether this association is causal. We undertook large-scale human genetic analyses to address this question.

**Methods and Findings:**

Genome-wide studies of BCAA levels in 16,596 individuals revealed five genomic regions associated at genome-wide levels of significance (*p <* 5 × 10^−8^). The strongest signal was 21 kb upstream of the *PPM1K* gene (beta in standard deviations [SDs] of leucine per allele = 0.08, *p* = 3.9 × 10^−25^), encoding an activator of the mitochondrial branched-chain alpha-ketoacid dehydrogenase (BCKD) responsible for the rate-limiting step in BCAA catabolism. In another analysis, in up to 47,877 cases of type 2 diabetes and 267,694 controls, a genetically predicted difference of 1 SD in amino acid level was associated with an odds ratio for type 2 diabetes of 1.44 (95% CI 1.26–1.65, *p* = 9.5 × 10^−8^) for isoleucine, 1.85 (95% CI 1.41–2.42, *p* = 7.3 × 10^−6^) for leucine, and 1.54 (95% CI 1.28–1.84, *p* = 4.2 × 10^−6^) for valine. Estimates were highly consistent with those from prospective observational studies of the association between BCAA levels and incident type 2 diabetes in a meta-analysis of 1,992 cases and 4,319 non-cases. Metabolome-wide association analyses of BCAA-raising alleles revealed high specificity to the BCAA pathway and an accumulation of metabolites upstream of branched-chain alpha-ketoacid oxidation, consistent with reduced BCKD activity. Limitations of this study are that, while the association of genetic variants appeared highly specific, the possibility of pleiotropic associations cannot be entirely excluded. Similar to other complex phenotypes, genetic scores used in the study captured a limited proportion of the heritability in BCAA levels. Therefore, it is possible that only some of the mechanisms that increase BCAA levels or affect BCAA metabolism are implicated in type 2 diabetes.

**Conclusions:**

Evidence from this large-scale human genetic and metabolomic study is consistent with a causal role of BCAA metabolism in the aetiology of type 2 diabetes.

## Abstract 2

Claudia Langenberg and colleagues show that high circulating branched chain amino acids associate with future risk of type 2 diabetes.

## Author Summary

**Why Was This Study Done?:**

- Higher circulating levels of isoleucine, leucine, and valine, i.e., the branched-chain amino acids (BCAAs), are strongly associated with the risk of future type 2 diabetes.
- It is not known if this association reflects a causal relationship.
- It is important to assess the aetiologic nature of this relationship. If it is causal, then intervening on BCAA levels or metabolism may reduce the risk of diabetes.

**What Did the Researchers Do and Find?:**

- We used a human genetics framework known as “Mendelian randomisation” to study this question. Mendelian randomisation postulates that if a biomarker is causally implicated in a disease, then genetic variants specifically associated with that biomarker should also be associated with the disease.
- In a meta-analysis of 1,992 incident cases of type 2 diabetes and 4,319 non-cases, we found strong associations between higher levels of each of the BCAAs and a higher risk of type 2 diabetes.
- In a genome-wide meta-analysis of 16,596 individuals, we identified five genomic regions where common genetic variants were associated with BCAA levels.
- In a meta-analysis of genetic association studies including 47,877 cases of type 2 diabetes and 267,694 controls, a genetically predicted difference of one standard deviation in amino acid level was associated with an odds ratio of type 2 diabetes of 1.44 (95% confidence interval 1.26–1.65) for isoleucine, 1.85 (1.41–2.42) for leucine, and 1.54 (1.28–1.84) for valine.

**What Do These Findings Mean?:**

- Evidence from this large-scale human genetic and metabolomic study is consistent with a causal role of BCAA metabolism in the aetiology of type 2 diabetes.
- Possible limitations of the study included the possibility of non-specific associations of the genetic variants included in the study (i.e., “pleiotropy”) and the relatively low proportion of heritability in BCAA levels explained by the identified genetic variants.

## Introduction

Early evidence of impaired branched-chain amino acid (BCAA) metabolism in insulin resistance and obesity dates back to more than 40 years ago [1]. More recently, in investigations of the human metabolome, multiple research groups have described associations of higher levels of isoleucine, leucine, and valine with insulin resistance, obesity [2], and a higher risk of future type 2 diabetes [3]. These associations have since been replicated in several studies [4–6] and are corroborated by a growing body of experimental evidence [2,7–10]. However, it remains unknown whether BCAA metabolism is causally implicated in type 2 diabetes [7] or whether observational associations simply reflect reverse causality, whereby differences in BCAA levels are a consequence of pathophysiological processes that precede the development of the disease [11].

Genetic approaches to causality assessment provide an opportunity for rapid and cost-effective prioritisation of targets for interventional studies [12–20]. Therefore, genetic variants associated with BCAA levels can be used to study the aetiologic links of the BCAA metabolic pathway with type 2 diabetes. A previous smaller-scale investigation that was limited to one genetic variant associated with 3-methyl-2-oxovalerate, a by-product of BCAA metabolism, was inconclusive [21].

Therefore, in this study, we used genome-wide association studies (GWASs) coupled with large-scale metabolomic measurements to investigate the aetiologic relationship between BCAA metabolism and type 2 diabetes, an issue of high clinical and public health relevance due to the strength of the associations between BCAA levels and type 2 diabetes [2,3] and the global burden of this condition [22,23].

## Methods

### Study Design

**Fig 1:**
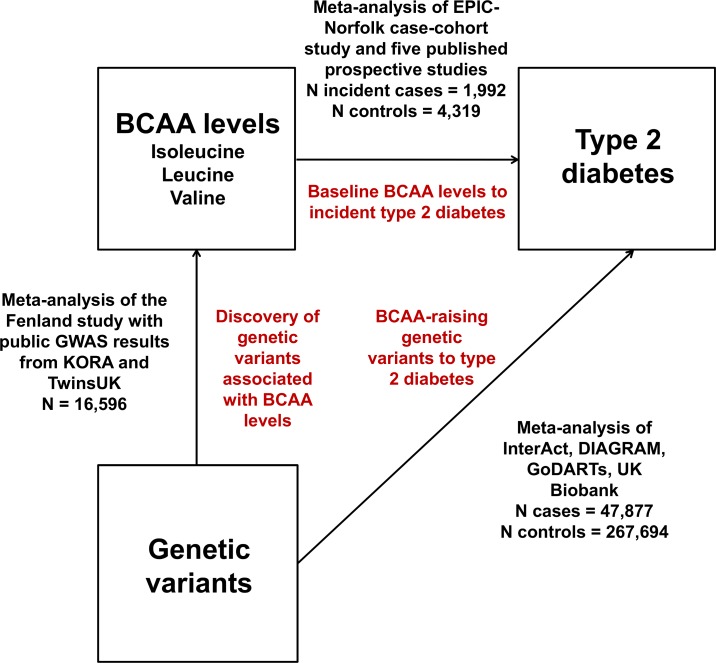
Design of the study. DIAGRAM, DIAbetes Genetics Replication And Meta-analysis.

We adopted a Mendelian randomisation approach to evaluate possible causal relationships between BCAA metabolism and type 2 diabetes (Fig 1). Inherited DNA variants are randomly assigned during meiosis and remain fixed throughout the lifetime. Therefore, they are unlikely to be affected by disease processes (i.e., reverse causality) and non-genetic confounding. Mendelian randomisation exploits these properties in order to estimate the relationship of modifiable risk factors with disease outcomes without the limitations of traditional observational epidemiology [12–15]. This approach postulates that if a biomarker is causally implicated in a disease, then genetic variants associated with that biomarker (but not other disease risk factors) should be associated with the disease in the direction predicted by observational analyses [12–15].

Therefore, we conducted meta-analyses of (a) GWASs of plasma levels of isoleucine, leucine, and valine; (b) studies of the associations of BCAA-raising genetic variants with type 2 diabetes; and (c) prospective studies of the associations of baseline isoleucine, leucine, and valine levels with incident type 2 diabetes.

The investigations described in this study were approved by local ethical committees, and participants provided their informed consent.

### Participating Studies

We performed GWASs of the plasma levels of isoleucine, leucine, and valine in the Fenland study (*n* = 9,237; S1 Text; S1 Table). Fenland GWAS results were then meta-analysed with publicly available results from a meta-analysis of the KORA and TwinsUK studies [24]. The total sample size of the genome-wide meta-analysis was 16,596 individuals.

We estimated the association with type 2 diabetes of the lead single nucleotide polymorphism (SNP) at each BCAA-associated genomic locus. We meta-analysed SNP association results from the DIAbetes Genetics Replication And Meta-analysis [25] and the EPIC-InterAct [26], GoDARTs [21], and UK Biobank studies [27] (S1 Text; S2 Table). The total sample size of this analysis was 47,877 type 2 diabetes cases and 267,694 controls.

We conducted a systematic review of the literature of prospective studies of the association of BCAA levels and incident type 2 diabetes (S1 Text). The results of five prospective population-based studies [3–6] were meta-analysed with the unpublished results of the EPIC-Norfolk case-cohort study using fixed effect models (S3 Table) [28]. The *I*-squared statistic was used to quantify heterogeneity. In the EPIC-Norfolk study, the association of metabolite levels with incident type 2 diabetes was estimated using multivariable Cox proportional hazards regression with Prentice weighting and robust standard errors. The total sample size of the observational analysis was 1,992 incident cases of type 2 diabetes and 4,319 non-cases.

### Metabolite Measurements

In the Fenland study, BCAA levels were measured using liquid chromatography coupled with tandem mass spectrometry (S1 Fig; AbsoluteIDQ p180 Kit, Biocrates Life Sciences [29]). In the KORA and TwinsUK studies, BCAA levels were measured as part of untargeted metabolomic measurements using gas chromatography mass spectrometry or ultra-performance liquid chromatography coupled with tandem mass spectrometry (UPLC-MS/MS) [24]. In the EPIC-Norfolk study, BCAA levels were measured by UPLC-MS/MS (S1 Fig; DiscoveryHD4 platform, Metabolon [30]). This platform also measured an additional 15 metabolites related to BCAA metabolism. In the Southall And Brent REvisited Study (SABRE), the levels of BCAAs were measured by nuclear magnetic resonance at 0 and 120 min during the course of an oral glucose tolerance test (OGTT) [31,32].

### Genome-Wide Association Analyses

Genome-wide association analyses were conducted using SNPTEST v2 [33], and study results were meta-analysed using METAL [34], which was chosen as the analysis software because studies included in the meta-analysis were focused on individuals of European ancestry. All genetic association results are reported per allele and assuming an additive effect. In the meta-analysis of GWASs of BCAA levels, we meta-analysed *Z*-scores rather than betas and standard errors in order to minimise the influence of the different platforms used to measure amino acid levels in participating studies. The conventional threshold for genome-wide statistical significance (*p* < 5 × 10^−8^) was used to identify associated loci. The SNP with the lowest *p*-value within a 1 million base-pair window was chosen as the lead SNP at a given genomic locus.

### Variance Explained and Genetic Correlation Analyses

For each amino acid, we estimated the variance in amino acid level explained by the identified lead SNPs using linear regression models in the Fenland study. We used the BOLT-REML algorithm of the BOLT-LMM v2.2 software [35] to estimate chip-based heritability. This was estimated directly from variants captured across the genome on the genome-wide genotyping chip. We also used estimates of family-based genetic heritability from twin studies from Shin and colleagues [24]. We calculated the proportion of heritability explained by the lead SNPs by dividing the explained variance estimates by the familial and chip-based heritability estimates. Using genome-wide association results and LD score regression [36], we estimated genetic correlations of BCAA levels with type 2 diabetes, continuous glycaemic traits (fasting glucose, fasting insulin, glucose at 2 h in a 75-g OGTT, HbA_1c_, HOMA-B, and HOMA-IR), and anthropometric traits (body mass index [BMI] and waist-to-hip ratio) on the LD Hub platform (http://ldsc.broadinstitute.org/#; accessed 6 October 2016) [37]. Isoleucine estimates were not generated by the software due to low levels of genetic heritability estimated by the LD Hub platform.

### Mendelian Randomisation Analysis

We estimated the association between a genetically predicted difference of 1 standard deviation (SD) in isoleucine, leucine, or valine plasma level and type 2 diabetes risk. For each of the BCAAs, we constructed weighted genetic risk scores including the lead SNP (independent genetic variants analysis) or genome-wide significant SNPs in imperfect linkage disequilibrium (*r* ^2^ < 0.8 for all pairwise SNP comparisons; correlated genetic variants analysis) at each locus identified by the genome-wide meta-analysis for that metabolite. Fenland study weights were used to scale the effect of each genetic score to 1 SD. We used Fenland study weights as this was the largest study and we had access to individual-level data, allowing the standardisation of genetic effect estimates. Lead SNPs (nearest gene) included in the genetic scores used in the independent variants analysis were as follows: rs7678928 (*PPM1K*), rs75950518 (*DDX19A*), rs58101275 (*TRMT61A*), and rs1420601 (*CBLN1*) for isoleucine; rs1440581 (*PPM1K*) for leucine; and rs1440581 (*PPM1K*) for valine. Analyses of correlated genetic variants modelled the estimates of 12 SNPs at four loci (*PPM1K*, *DDX19A*, *TRMT61A*, and *CBLN1*) for isoleucine, seven SNPs at the *PPM1K* locus for leucine, and eight SNPs at the *PPM1K* locus for valine (details in S1 Text).

We combined estimates of “SNP to metabolite” and “SNP to type 2 diabetes” associations to calculate estimates of each “genetically predicted metabolite level to type 2 diabetes” association [13,14]. Estimates of multiple SNPs contributing to a given genetic score were pooled using an inverse-variance-weighted method [13,14]. For the analysis of correlated genetic variants, estimates were pooled with a weighted generalised linear regression method that accounts for the correlation between genetic variants [13]. The correlation values were obtained using the SNAP software [38]. Results were scaled to represent the odds ratio [OR] per 1-SD genetically predicted difference in amino acid level. Given that Mendelian randomisation assumes no pleiotropic effect beyond that on the risk factor of interest (i.e., BCAA levels), we excluded the lead SNP (rs1260326) at the known pleiotropic [39–43] *GCKR* locus from the isoleucine genetic score (Section 1 of S2 Text).

In order to assess the specificity of the genetic scores, we performed large-scale association testing of BCAA-raising alleles with the levels of blood metabolites: 175 metabolites in the Fenland study, 453 metabolites in KORA and TwinsUK, and 18 metabolites of the BCAA pathway in the EPIC-Norfolk study (S1 Text). We also investigated the association of BCAA-raising alleles with cardiometabolic traits in large-scale GWAS meta-analyses of up to 328,036 individuals [39–43].

### Type 2 Diabetes Disease Mechanisms and BCAA Metabolism

We investigated other potential links between BCAA levels and type 2 diabetes. We tested the association with BCAA levels of a genetic predisposition to higher BMI, greater insulin resistance, and impaired insulin secretion using previously validated unweighted genetic scores for these traits [44–46] (S4 Table). Using linear regression models, we estimated the association of fasting insulin with BCAA levels during the course of an OGTT in the SABRE study.

### Expression of *PPM1K* in Muscle Biopsies

We investigated changes in levels of *PPM1K* gene expression in skeletal muscle during the course of an OGTT. Fifty age-matched men with either normal glucose tolerance (*n* = 25) or type 2 diabetes (*n* = 25) were recruited at the Karolinska Institutet, Sweden. Following an overnight fast, a skeletal muscle biopsy was taken from the vastus lateralis muscle under local anaesthesia with a Weil-Blakesley conchotome tong instrument (Agntho’s). Participants ingested a standardised solution containing 75 g of glucose, and 2 h later a second biopsy was taken from the vastus lateralis of the contralateral leg. Biopsies were frozen immediately and stored in liquid nitrogen until processed. Total RNA was extracted from biopsies using the mirVana miRNA Isolation Kit (Thermo Fisher). Equal amounts of total RNA were used to synthesize cDNA using random primers and the High Capacity cDNA Reverse Transcription Kit (Thermo Fisher). Quantitative PCR was performed with a ViiA 7 Real-Time PCR System with Fast SYBR Green Master Mix (Thermo Fisher). The ViiA 7 Software (version 1.1) was used to determine threshold cycle (Ct) values, and relative gene expression was calculated with the comparative Ct method relative to a reference gene, with dCt being the difference in expression between the gene of interest and the reference gene. The most suitable reference genes were determined to be *GUSB*, *RPLP0*, and *TBP* (out of four measured genes) using the NormFinder algorithm (Multid Analyses) [47]. Statistical analysis was performed on dCt values since these were normally distributed.

### Statistical Analysis

Both Mendelian randomisation and observational association estimates are reported per 1-SD increase in metabolite level. Standardisation was necessary in light of the different platforms used to measure BCAA levels. Analyses were conducted using STATA v13.1 (StataCorp) and R (https://cran.r-project.org/). Further details of the methods used in this study are reported in S1 Text.

## Results

### Genome-Wide Association Studies of Isoleucine, Leucine, and Valine

**Fig 2:**
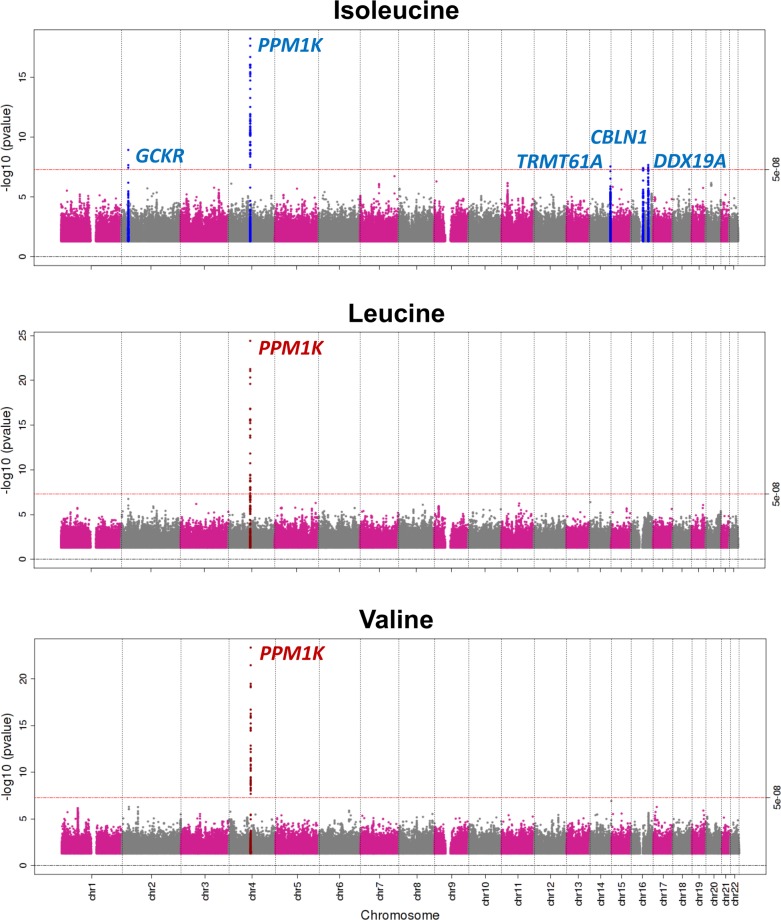
Manhattan plot of the association of genetic variants with the levels of branched-chain amino acids.

Genome-wide meta-analyses of 10.5 million genetic variants in 16,596 individuals revealed five independent genomic loci associated with BCAA levels at the genome-wide level of statistical significance (Table 1; Figs 2, S2 and S3). The strongest signal was 21 kb upstream of the *PPM1K* gene on Chromosome 4q22.1, a locus previously reported for BCAA levels [24,48] (Fig 2).

**Table 1:** Genetic variants associated with the levels of branched-chain amino acids in the genome-wide meta-analysis.

Metabolite	Locus	Lead SNP	Genomic Coordinate	Effect/Other Alleles	EAF	Beta (SE) of Metabolite Level per Allele	*p*-Value	Sample Size for Type 2 Diabetes, *N* Cases/*N* Controls	OR (95% CI) for Type 2 Diabetes per Allele	*p*-Value
**Isoleucine**	*PPM1K*	rs7678928	4:89222827	T/C	46%	0.09 (0.013)	5.6 × 10^−19^	25,208/209,575	1.03 (1.01–1.05)	0.0055
	*GCKR*	rs1260326	2:27730940	T/C	41%	0.06 (0.012)	1.1 × 10^−09^	47,877/267,694	0.94 (0.92–0.96)	3.9 × 10^−11^
	*DDX19A*	rs75950518	16:70378917	C/T	89%	0.11 (0.019)	2.1 × 10^−08^	13,037/152,713	1.05 (1.01–1.10)	0.016
	*TRMT61A*	rs58101275	14:104008420	G/A	79%	0.09 (0.015)	2.8 × 10^−08^	13,037/152,713	1.04 (1.01–1.08)	0.012
	*CBLN1*	rs1420601	16:49085649	C/T	40%	0.07 (0.013)	3.7 × 10^−08^	13,037/152,713	1.01 (0.98–1.04)	0.53
**Leucine**	*PPM1K*	rs1440581	4:89226422	C/T	53%	0.08 (0.013)	3.9 × 10^−25^	30,169/215,523	1.04 (1.02–1.07)	0.00034
**Valine**	*PPM1K*	rs1440581	4:89226422	C/T	53%	0.10 (0.013)	4.4 × 10^−24^	30,169/215,523	1.04 (1.02–1.07)	0.00034

Genomic coordinates are relative to human genome reference sequence hg19. Beta coefficients are in standardised units.

EAF, effect allele frequency; OR, odds ratio; SNP, single nucleotide polymorphism; SE, standard error.

*PPM1K* encodes the mitochondrial phosphatase that activates the branched-chain alpha-ketoacid dehydrogenase (BCKD) complex [49–51]. This catalytic complex is responsible for the rate-limiting step of BCAA catabolism, i.e., the irreversible oxidative decarboxylation of branched-chain alpha-ketoacids [49–51]. In addition to *PPM1K*, we identified four novel loci associated with isoleucine (Table 1; Fig 2). Lead SNPs at these loci were also associated with the levels of the other two BCAAs in a consistent direction, albeit not at the genome-wide level of significance (S5 Table).

We estimated that identified lead genetic variants explain 7.5%, 6.3%, and 5.3% of the chip-based heritability of isoleucine, leucine, and valine levels estimated in the Fenland study using BOLT-LMM [35] and 2.2%, 0.8%, and 1.2% of the family-based heritability of isoleucine, leucine, and valine levels estimated in twin studies [24], respectively.

### Association of BCAA-Raising Alleles with Type 2 Diabetes

**Fig 3:**
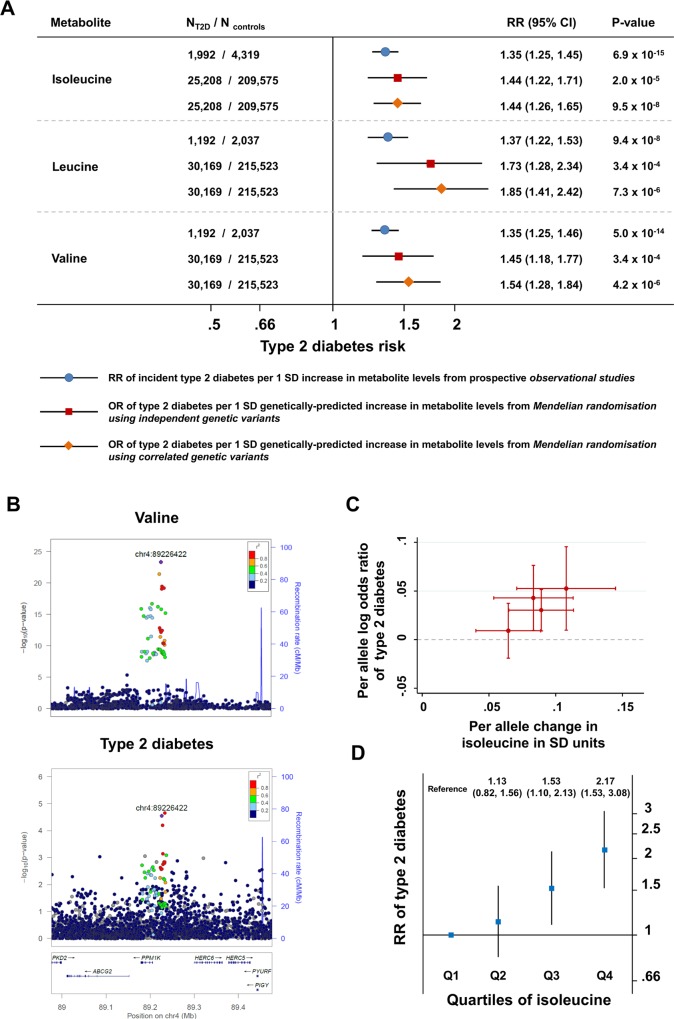
Genetically predicted or measured levels of the branched-chain amino acids and risk of type 2 diabetes. (A) Comparison of (i) the association of a difference of 1 SD in the levels of BCAAs at baseline with incident type 2 diabetes in prospective observational studies (bars with blue circles) and (ii) the association of a genetically predicted difference of 1 SD in BCAA levels with type 2 diabetes in genetic Mendelian randomisation studies (bars with red squares for analyses of independent genetic variants and bars with orange diamonds for analyses of correlated genetic variants). (B) Regional association plots for the association of variants at the *PPM1K* locus with valine (top; representative of the three BCAAs) and type 2 diabetes (bottom). Associations were characterised by a peak of signal upstream of the *PPM1K* gene, with the lead rs1440581 polymorphism and other polymorphisms in high linkage disequilibrium, as well as a peripheral signal overlaying the gene, with variants in lower linkage disequilibrium with the lead polymorphism. (C) Scatter plot of the association of the isoleucine-raising alleles included in the isoleucine genetic score with amino acid levels and with type 2 diabetes risk. (D) The association of the quartiles of isoleucine level at baseline with incident type 2 diabetes in the EPIC-Norfolk case-cohort study, the Framingham Offspring Study, and the Malmö Diet and Cancer Study (1,025 cases of incident type 2 diabetes and 1,182 controls). Error bars represent the 95% confidence intervals around the central estimates. BCAA, branched-chain amino acid; OR, odds ratio; RR, relative risk; SD, standard deviation; T2D, type 2 diabetes.

A genetic predisposition to a higher level of isoleucine, leucine, or valine was strongly associated with higher odds for type 2 diabetes (for isoleucine, OR 1.44, 95% CI 1.26–1.65, *p* = 9.5 × 10^−8^; for leucine, OR 1.85, 95% CI 1.41–2.42, *p* = 7.3 × 10^−6^; for valine, OR 1.54, 95% CI 1.28–1.84, *p* = 4.2 × 10^−6^; Fig 3A). Relative risk estimates from genetic analyses based on the identified genetic variants were similar to those from the prospective studies of the association between baseline amino acid levels and incident type 2 diabetes (Figs 3A, S4 and S5; Section 2 of S2 Text). For isoleucine, the results of the genetic analyses were almost identical when considering genetic variants at the *PPM1K* locus only or genetic variants at all four loci (S6 Table). At the *PPM1K* locus, both the lead SNPs in the BCAA association analyses, rs1440581 (*r* ^2^ = 0.88) and rs7678928 (*r* ^2^ = 0.70), were in linkage disequilibrium with the lead type 2 diabetes SNP at the locus, rs1975393 (Fig 3B). There was a dose-response relationship between the association with amino acid level and the relative increase in diabetes risk for the four lead SNPs that were used for the construction of the isoleucine genetic score (Fig 3C). Similarly, the association between isoleucine level and incident type 2 diabetes in prospective studies showed a graded dose-response relationship (Fig 3D; S7 Table).

### BCAA-Raising Alleles, Continuous Metabolic Traits, and Plasma Metabolites

**Fig 4:**
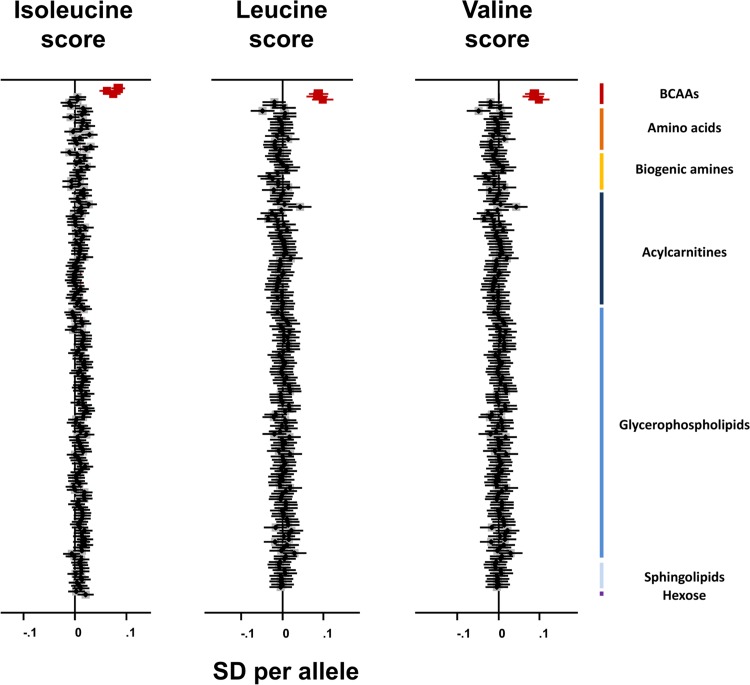
Association of branched-chain amino acid genetic scores with metabolites in the Fenland study. Associations of the BCAA scores with the BCAAs are highlighted in dark red. Error bars represent the 95% confidence interval around the central estimate.BCAA, branched-chain amino acid; SD, standard deviation.

The isoleucine, leucine, and valine genetic scores were specifically associated with the three BCAAs and not with any of the remaining 172 metabolites measured in the Fenland study (Fig 4). Consistent with these results, the rs1440581 SNP at *PPM1K* was associated with only eight out of 453 metabolites studied by Shin et al. [24], all of which belonged to the BCAA pathway (S8 Table). These results illustrate the high specificity to BCAA metabolism of these genetic scores.

The BCAA-raising genetic scores and their constituent SNPs were not associated with continuous metabolic traits in large-scale meta-analyses. There was an association of the isoleucine-raising allele of rs1420601 near *CBLN1* with higher BMI (S9 Table), but this did not affect the main analysis results (OR for type 2 diabetes after exclusion of rs1420601 = 1.50, 95% CI 1.25–1.80, *p* = 0.000013).

We assessed genome-wide genetic correlations of BCAA levels with type 2 diabetes and continuous metabolic traits and found statistically significant (*p <* 0.05) positive correlations of (a) leucine level with type 2 diabetes (*r*_genetic_ = 0.34, *p* = 0.0004), HbA_1c_ (*r*_genetic_ = 0.34, *p* = 0.0038), BMI (*r*_genetic_ = 0.25, *p* = 1.5 × 10^−5^), and waist-to-hip ratio (*r*_genetic_ = 0.29, *p* = 2.6 × 10^−6^) and of (b) valine level with type 2 diabetes (*r*_genetic_ = 0.54, *p* = 8.8 × 10^−5^), fasting insulin (*r*_genetic_ = 0.42, *p* = 0.013), BMI (*r*_genetic_ = 0.43, *p* = 1.8 × 10^−5^), waist-to-hip ratio (*r*_genetic_ = 0.47, *p* = 6.4 × 10^−6^), HOMA-B (*r*_genetic_ = 0.40, *p* = 0.016), and HOMA-IR (*r*_genetic_ = 0.45, *p* = 0.012).

### Type 2 Diabetes Disease Mechanisms and BCAA Metabolism

In the Fenland study, both higher BMI and higher fasting insulin level, a measure of fasting-state insulin resistance, were associated with higher levels of BCAAs (S10 Table). In addition, genetic predispositions to higher BMI and insulin resistance, but not to impaired insulin secretion, were associated with higher levels of BCAAs (S11 and S12 Tables; S6 Fig). The association of genetic predisposition to greater adiposity with high BCAA levels appeared to be mediated by insulin resistance (S11 Table). In the SABRE study, a glucose challenge resulted in a reduction of circulating BCAA levels. Individuals with higher fasting insulin showed a diminished reduction of all three BCAA levels in response to a glucose challenge (S7 Fig; S13 and S14 Tables). In muscle biopsies collected during an oral glucose challenge, the expression of *PPM1K* increased at 2 h in normoglycaemic individuals, but not in age-matched patients with type 2 diabetes (S8 Fig). These results are in line with what has been reported in previous investigations [52,53] and suggest that greater adiposity and impaired insulin sensitivity result in exposure to higher levels of BCAAs both in the fasting state and after glucose intake, which could be mediated at least in part by impaired BCKD activation.

### BCAA Pathway Analysis

**Fig 5:**
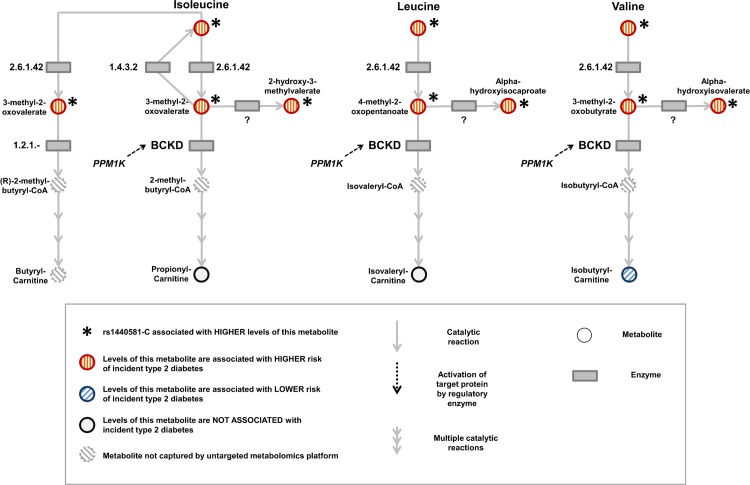
Schematic representation of the branched-chain amino acid pathway and associations with type 2 diabetes. BCKD, branched-chain alpha-ketoacid dehydrogenase.

Because BCAA-raising alleles were strongly associated with metabolites specific to the BCAA pathway, we hypothesized that studying their pattern of association might provide additional insights into the mechanisms affected by these genetic variants. Using untargeted metabolomics in up to 8,693 individuals, we investigated the association of BCAA-raising alleles with the levels of 18 metabolites involved in BCAA metabolism (Tables 2 and S15; Fig 5). BCAA-raising genetic variants were strongly associated with higher levels of the branched-chain alpha-ketoacids and other metabolites upstream of the irreversible branched-chain alpha-ketoacid dehydrogenation (Fig 5). Metabolites downstream of this important biochemical reaction were largely unaffected by BCAA-raising alleles (Tables 2 and S15; Fig 5).

**Table 2:** BCAA pathway associations.

Position in the BCAA Pathway	Category	Metabolite	Beta (SE) for Metabolite per rs1440581-C Allele	*p*-Value	RR (95% CI) of Type 2 Diabetes per 1-SD Increase in Metabolite Level	*p*-Value
**Upstream of BCKD**	BCAA	Isoleucine	0.09 (0.02)	6.0 × 10^−8^	1.31 (1.11–1.54)	0.0012
		Leucine	0.11 (0.01)	8.2 × 10^−19^	1.22 (1.03–1.43)	0.018
		Valine	0.12 (0.02)	4.6 × 10^−12^	1.34 (1.12–1.61)	0.0013
	BCKA	3-methyl-2-oxovalerate	0.13 (0.01)	1.5 × 10^−19^	1.62 (1.35–1.95)	3.1 × 10^−7^
		3-methyl-2-oxobutyrate	0.13 (0.02)	3.3 × 10^−14^	1.60 (1.33–1.92)	5.8 × 10^−7^
		3-hydroxy-2-ethylpropionate	0.12 (0.02)	2.4 × 10^−13^	1.40 (1.21–1.63)	1.2 × 10^−5^
	BCKA-derived	2-hydroxy-3-methylvalerate	0.11 (0.04)	0.0042	1.36 (1.18–1.58)	3.4 × 10^−5^
		Alpha-hydroxyisovalerate	0.09 (0.02)	8.8 × 10^−9^	1.22 (1.05–1.41)	0.0098
		Alpha-hydroxyisocaproate	0.12 (0.04)	0.00066	1.22 (1.03–1.45)	0.025
**Downstream of BCKD**	Other	3-hydroxyisobutyrate	0.01 (0.04)	0.71	1.32 (1.13–1.54)	0.00047
	Acylcarnitine	Propionylcarnitine	−0.04 (0.02)	0.010	1.15 (0.98–1.34)	0.078
		Isovalerylcarnitine	−0.01 (0.02)	0.42	1.13 (0.97–1.32)	0.11
		Isobutyrylcarnitine	0.02 (0.02)	0.13	0.82 (0.70–0.96)	0.014

The table reports the association of a genetic predisposition to higher BCAA levels with the levels of other metabolites in the BCAA pathway. The table also reports the association of higher levels of metabolites in the BCAA pathway with the risk of incident type 2 diabetes. Beta coefficients are in standardised units.

BCAA, branched-chain amino acid; BCKA, branched-chain alpha-ketoacid; BCKD, branched-chain alpha-ketoacid dehydrogenase; RR, relative risk; SE, standard error; SD, standard deviation.

Higher levels of branched-chain alpha-ketoacids and other metabolites upstream of BCKD action were also strongly associated with a higher risk of incident type 2 diabetes in the EPIC-Norfolk case-cohort study, whereas the associations of downstream metabolites were inconsistent (Tables 2 and S15; Fig 5).

## Discussion

In this large-scale human genetic study, BCAA-raising polymorphisms identified with a genome-wide approach were associated with a higher risk of type 2 diabetes. Our findings suggest that mechanisms leading to impaired BCAA metabolism are implicated in the pathophysiology of type 2 diabetes. Given the strong and specific association with the BCAA pathway observed after testing more than 500 metabolic phenotypes, it is unlikely that the identified genetic variants affect type 2 diabetes risk via mechanisms outside of this metabolic pathway. The relative increase in diabetes risk associated with these genetic variants appeared to be proportional to the size of the association with amino acid levels, which would further support a possible causal link. The diabetes risk increase estimated in genetic analyses was also consistent with the direction and magnitude of association between baseline amino acid levels and incident diabetes in observational prospective studies.

A genetic predisposition to insulin resistance was associated with higher plasma BCAA levels, suggesting that previously reported associations between genetic susceptibility to higher BMI and BCAA levels [11] may be mediated by insulin resistance mechanisms. Consistent with recent studies in mice [54], the expression of *PPM1K* in muscle biopsies during an oral glucose challenge failed to increase in people with type 2 diabetes, suggesting that the link between insulin resistance and higher BCAA levels may be partly mediated by impaired BCKD activation. Therefore, part of the contribution of insulin resistance mechanisms to type 2 diabetes may be exerted via impaired BCAA metabolism.

In metabolome-wide investigations of BCAA-raising alleles, we found evidence of an accumulation of BCAAs and BCAA-derived metabolites upstream of the oxidative dehydrogenation of branched-chain alpha-ketoacids. This reaction is the irreversible and rate-limiting step in BCAA metabolism, catalysed by the mitochondrial BCKD complex [55]. The pattern of association observed in this study mirrors that observed in maple syrup urine disease (MSUD), an inborn error of metabolism caused by rare loss-of-function mutations in genes encoding components of the BCKD complex [55] or its regulatory phosphatase [56]. In our GWAS of BCAA levels, the strongest association signal was located 21 kb upstream of the *PPM1K* gene, which encodes the mitochondrial phosphatase that activates BCKD [49–51]. Loss-of-function mutations of *PPM1K* in humans [56] or the knock-out of its ortholog *Ppm1k* in mice models [35] results in impaired BCKD activity and high levels of BCAAs and branched-chain alpha-ketoacids, a pattern that resembles that observed for common *PPM1K* genetic variants in our study. Therefore, it is plausible that the genetic variants identified in this study act by impairing the catabolism of BCAAs, hence leading to higher circulating levels of these amino acids. We found that levels of all metabolites accumulated upstream of BCKD action were associated with incident type 2 diabetes. This further supports the hypothesis that reduced BCKD activity could be one of the mechanistic links between BCAA metabolism and type 2 diabetes.

Our findings have public health and clinical implications as they indicate that modulation of BCAA metabolism may impact diabetes risk. The activity of BCKD is a major determinant of the rate of BCAA catabolism and is amenable to modulation by pharmacological intervention [57–62]. Improving insulin sensitivity may also ameliorate BCAA metabolism, resulting in reduced diabetes risk. This approach is supported by the well-described reduction of amino acid levels following the administration or secretion of insulin [63,64], by our finding of higher BCAA levels in individuals with a genetic predisposition to insulin resistance, and by the observation of reduced BCAA levels following insulin-sensitising interventions [65–67].

Future studies will clarify the molecular mechanisms linking impaired BCAA metabolism and increased risk of type 2 diabetes. The lack of a strong association between BCAA-raising alleles and continuous metabolic traits in publicly available GWAS meta-analyses possibly reflects a lack of statistical power or the unavailability of data about specific glycaemic traits altered by these alleles, similar to several other type 2 diabetes risk variants [25]. We found positive genetic correlations of BCAA levels with type 2 diabetes and several glycaemic and anthropometric traits, which points to shared genetic determinants between BCAA levels and greater adiposity, hyperinsulinaemia, and hyperglycaemia.

A growing body of evidence from human, cellular, and animal models is beginning to shed light on the possible mechanisms linking BCAA metabolism and diabetes risk [2,7,8,68]. Emerging mechanistic explanations include a synergistic interference of BCAAs and lipids with the response of peripheral tissues to insulin [8]. In animal feeding studies, BCAA supplementation requires the background of a high-fat diet to promote insulin resistance [8]. The rs1440581-C variant, i.e., the lead BCAA-raising allele in our genome-wide association analysis, was found to be associated with less weight loss and insulin sensitisation following a calorie-restricted, high-fat diet in a dietary intervention study in humans [69]. Recent research has shown that higher BCAA levels are associated with a gut microbiome pattern characterised by enriched BCAA biosynthetic potential, including *Prevotella copri* and *Bacteroides vulgatus* species, pointing to a possible role of the gut flora in the relationship between BCAA levels and insulin resistance [70]. BCAAs and their by-products have also been linked to beta-cell dysfunction [7–9]. The knock-down of *PPM1K* in clonal INS-1 cell lines has been shown to impair glucose-stimulated insulin secretion [9]. It has also been suggested that a chronic exposure to high levels of BCAAs may result in a constant hyperinsulinaemic response, eventually leading to beta-cell exhaustion [8]. The increased oxidative stress associated with the accumulation of BCAA-derived alpha-ketoacids is consistent with the link between superoxide generation and beta-cell dysfunction [71,72].

Another aspect to be elucidated is whether patients with MSUD have altered glycaemic metabolism. Alterations of glucose metabolism were not reported as a clinical feature in a review of the phenotype of the disease [55]. However, Mogos et al. reported hypoglycaemia in a case of MSUD, which could perhaps be consistent with the hypothesis of BCAA-mediated hyperinsulinaemia [73]. MSUD can be fatal early in life, is often associated with severe cognitive impairment, and is managed with a tightly controlled therapeutic diet [55]. Therefore, MSUD may not be an optimal model to study the risk of a late-life disease with important dietary determinants such as type 2 diabetes.

Limitations of the genetic approach used in this study affect its interpretation. Mendelian randomisation assumes that the genetic variants used as instruments are associated with the disease exclusively via the risk factor of interest. For this reason, we excluded the pleiotropic *GCKR* locus from analysis. We also assessed the association of genetic variants with more than 500 phenotypes, without finding evidence of pleiotropy. While this reduces the possibility that pleiotropy has influenced our findings, we cannot entirely exclude the possibility of pleiotropic associations. Similar to other complex traits, the genetic variants identified in this study explain only a fraction of the heritability of BCAA levels. Therefore, it is possible that only some of the mechanisms that increase BCAA levels or affect their metabolism are implicated in type 2 diabetes.

While our genetic approach investigated the relationships between circulating BCAA levels and diabetes risk, BCAA metabolism is a complex biological phenomenon closely linked to other metabolic pathways. It is plausible that changes in this metabolic pathway may influence diabetes risk only in a certain metabolic context. Some suggestions come from the emerging biology of the glucokinase regulatory protein (GCKR), the genetic variants of which had to be excluded from our study for methodological reasons. GCKR is known to switch liver metabolism from production of glucose towards that of triglycerides, in particular triglycerides with lower carbon content and double bonds [74,75]. These lipid species are associated with higher diabetes incidence in observational studies [76]. Therefore, the activation of specific pathways (such as those that increase circulating BCAAs or short-chain, highly saturated triglycerides) may typically increase type 2 diabetes risk, except when such activation is caused by GCKR. These findings suggest that (a) the mechanisms by which the concentrations of these biomarkers are raised, not their higher concentrations per se, are implicated in higher metabolic risk and (b) higher BCAA levels might be tolerated as long as other protective pathways are activated. Larger-scale genetic studies and randomised controlled trials will help to further qualify the causal nature of the relationship between BCAA metabolism and type 2 diabetes risk.

### Conclusions

In this study, BCAA-raising polymorphisms identified with a genome-wide approach were associated with a higher risk of type 2 diabetes, consistent with a causal role of BCAA metabolism in the aetiology of this common complex disease.

## Supporting Information

S1 PRISMA Checklist (DOCX)

S1 Fig Distribution of branched-chain amino acid levels in the cohorts included in the study. (DOCX)

S2 Fig Regional plots of associated loci and quantile-quantile plots of the genome-wide meta-analyses of leucine and valine. (DOCX)

S3 Fig Regional plots of associated loci and quantile-quantile plot of the genome-wide meta-analysis of isoleucine. (DOCX)

S4 Fig Workflow of the systematic review of the literature about the observational association between BCAA levels and incident type 2 diabetes. (DOCX)

S5 Fig Meta-analysis of the observational association between baseline BCAA levels and incident type 2 diabetes. (DOCX)

S6 Fig Association of body mass index, insulin resistance, and impaired insulin secretion genetic scores with the levels of branched-chain amino acids in the Fenland, KORA, and TwinsUK studies. (DOCX)

S7 Fig Levels of branched-chain amino acids during the course of an oral glucose tolerance test in the SABRE study. (A) The change of amino acid levels during the OGTT, calculated as 120-min levels minus fasting levels. (B) The change by quartiles of fasting insulin. *p*-Values are from linear regression models in which fasting insulin (continuous) was the exposure, change in amino acid levels was the outcome, and age, sex, BMI, and fasting amino acid levels were the covariates.
(DOCX)

S8 Fig Change in *PPM1K* gene expression during the course of an oral glucose challenge in men with type 2 diabetes and age-matched normoglycaemic controls. (A) The relative expression of *PPM1K* in skeletal muscle biopsies taken at baseline and 120 min after an oral glucose challenge in the two groups. Bars represent the mean, and error bars the standard error of the mean. (B) The difference in *PPM1K* expression levels between 120 min and baseline in the two groups. Full circles represent the mean difference, and error bars their 95% confidence intervals. The *p*-values for the difference, calculated using two-tailed ANOVA with Šidák correction for multiple testing, were *p* = 0.013 in normoglycaemic controls and *p* = 0.55 in type 2 diabetes patients.
(DOCX)

S9 Fig Associations with type 2 diabetes and metabolite levels of the isoleucine genetic score with and without the *GCKR* locus. The figure represents the association with type 2 diabetes (A) and with metabolite levels in the Fenland study (B) of the isoleucine genetic score with and without the *GCKR* locus.
(DOCX)

S1 Table Characteristics of the cohorts included in the genome-wide meta-analysis of branched-chain amino acid levels. (DOCX)

S2 Table Characteristics of the cohorts included in the analysis of the association of BCAA-raising alleles with type 2 diabetes. (DOCX)

S3 Table Characteristics of the studies included in the meta-analysis of the observational association between baseline levels of the branched-chain amino acids and incident type 2 diabetes. (DOCX)

S4 Table Genetic scores for increased body mass index, insulin resistance, and impaired insulin secretion. (DOCX)

S5 Table Association of the lead genetic variant at each locus with the level of each of the branched-chain amino acids. (DOCX)

S6 Table Sensitivity analyses of the association of the isoleucine genetic score with type 2 diabetes. (DOCX)

S7 Table Association of baseline isoleucine level with incident type 2 diabetes by quartile of isoleucine level. The outcome of all the analyses was incident type 2 diabetes.
(DOCX)

S8 Table Association of the rs1440581 variant at the *PPM1K* locus with metabolites in the study by Shin et al. [24]. Only metabolites significantly associated after correction for multiple testing are reported.
(DOCX)

S9 Table Association of BCAA-raising genetic variants with continuous metabolic traits in large-scale meta-analyses. (DOCX)

S10 Table Association of fasting insulin and body mass index with branched-chain amino acid levels in the Fenland study. (DOCX)

S11 Table Association of body mass index, insulin resistance, and impaired insulin secretion genetic scores with branched-chain amino acids in the Fenland, TwinsUK, and KORA studies. (DOCX)

S12 Table Association of the insulin resistance genetic score with branched-chain amino acids in the TwinsUK and KORA studies. (DOCX)

S13 Table Levels of branched-chain amino acids during the course of an oral glucose tolerance test in the SABRE study. (DOCX)

S14 Table Association of fasting insulin with levels of branched-chain amino acids (i.e., difference of 120 min and 0 min) after a glucose challenge. (DOCX)

S15 Table Branched-chain amino acid pathway analysis. The table reports the association of BCAA genetic scores with all measured BCAA-related metabolites in the EPIC-Norfolk, TwinsUK, and KORA studies. The table also reports the association of BCAA-related metabolites with incident type 2 diabetes in the EPIC-Norfolk case-cohort study.
(DOCX)

S1 Text Supplementary methods. (DOCX)

S2 Text Supplementary results. (DOCX)

## Abbreviations

BCAA: branched-chain amino acid
BCKD: branched-chain alpha-ketoacid dehydrogenase
BMI: body mass index
Ct: threshold cycle
GWAS: genome-wide association study
MSUD: maple syrup urine disease
OGTT: oral glucose tolerance test
SABRE: Southall And Brent REvisited Study
SD: standard deviation
SNP: single nucleotide polymorphism
