# Supplementary material for: Genetic Predisposition to an Impaired Metabolism of the Branched-Chain Amino Acids and Risk of Type 2 Diabetes: A Mendelian Randomisation Analysis
Source: PLoS Med. 2016 Nov 29;13(11):e1002179. doi: 10.1371/journal.pmed.1002179 (PMC5127513; doi:10.1371/journal.pmed.1002179)
Supplement: S2 Fig — (DOCX) [file pmed.1002179.s003.docx]

**S2 Fig. Regional plots of associated loci and quantile-quantile plots of the genome-wide meta-analyses of leucine and valine.**
