# Supplementary material for: Genetic Predisposition to an Impaired Metabolism of the Branched-Chain Amino Acids and Risk of Type 2 Diabetes: A Mendelian Randomisation Analysis
Source: PLoS Med. 2016 Nov 29;13(11):e1002179. doi: 10.1371/journal.pmed.1002179 (PMC5127513; doi:10.1371/journal.pmed.1002179)
Supplement: S6 Fig — (DOCX) [file pmed.1002179.s007.docx]

**S6 Fig. Association of body mass index, insulin resistance and impaired insulin secretion genetic scores with the levels of branched chain amino acids in the Fenland, KORA and Twins UK studies.**
