# Supplementary material for: Genetic Predisposition to an Impaired Metabolism of the Branched-Chain Amino Acids and Risk of Type 2 Diabetes: A Mendelian Randomisation Analysis
Source: PLoS Med. 2016 Nov 29;13(11):e1002179. doi: 10.1371/journal.pmed.1002179 (PMC5127513; doi:10.1371/journal.pmed.1002179)
Supplement: S7 Fig — (A) The change of amino acid levels during the OGTT, calculated as 120-min levels minus fasting levels. (B) The change by quartiles of fasting insulin. p-Values are from linear regression models in which fasting insulin (continuous) was the exposure, change in amino acid levels was the outcome, and age, sex, BMI, and fasting amino acid levels were the covariates. (DOCX) [file pmed.1002179.s008.docx]

**S7 Fig. Levels of branched chain amino acids during the course of an oral glucose tolerance test (OGTT) in the SABRE study.** Panel A reports the change of amino acid levels during the OGTT, calculated as 120 minute levels – fasting levels. Panel B reports the change by quartiles of fasting insulin. P-values are from linear regression models in which fasting insulin (continuous) was the exposure, change in amino acid levels was the outcome, and age, sex, body mass index and fasting amino acid levels were the covariates.
