# Supplementary material for: Genetic Predisposition to an Impaired Metabolism of the Branched-Chain Amino Acids and Risk of Type 2 Diabetes: A Mendelian Randomisation Analysis
Source: PLoS Med. 2016 Nov 29;13(11):e1002179. doi: 10.1371/journal.pmed.1002179 (PMC5127513; doi:10.1371/journal.pmed.1002179)
Supplement: S8 Fig — (A) The relative expression of PPM1K in skeletal muscle biopsies taken at baseline and 120 min after an oral glucose challenge in the two groups. Bars represent the mean, and error bars the standard error of the mean. (B) The difference in PPM1K expression levels between 120 min and baseline in the two groups. Full circles represent the mean difference, and error bars their 95% confidence intervals. The p-values for the difference, calculated using two-tailed ANOVA with Šidák correction for multiple testing, were p = 0.013 in normoglycaemic controls and p = 0.55 in type 2 diabetes patients. (DOCX) [file pmed.1002179.s009.docx]

**S8 Fig. Change in *PPM1K* gene expression during the course of an oral glucose challenge in men with type 2 diabetes and age-matched normoglycaemic controls.** Panel A shows the relative expression of *PPM1K* in skeletal muscle biopsies taken at baseline and 120 minutes after an oral glucose challenge in the two groups. Bars represent mean and error bars the standard error of the mean. Panel B shows the difference in *PPM1K* expression levels between 120 minutes and baseline in the two groups. Full circles represent the mean difference and error bars their 95% confidence intervals. The p-values for the difference, calculated using two-tailed ANOVA with Šidák correction for multiple testing were p=0.013 in normoglycaemic controls and p=0.55 in type 2 diabetes patients.
