# Supplementary material for: Genetic Predisposition to an Impaired Metabolism of the Branched-Chain Amino Acids and Risk of Type 2 Diabetes: A Mendelian Randomisation Analysis
Source: PLoS Med. 2016 Nov 29;13(11):e1002179. doi: 10.1371/journal.pmed.1002179 (PMC5127513; doi:10.1371/journal.pmed.1002179)
Supplement: S9 Fig — The figure represents the association with type 2 diabetes (A) and with metabolite levels in the Fenland study (B) of the isoleucine genetic score with and without the GCKR locus. (DOCX) [file pmed.1002179.s010.docx]

**S9 Fig. Associations with type 2 diabetes and metabolite levels of the isoleucine genetic score with and without the *GCKR* locus.** The figure represents the association with type 2 diabetes (A) and with metabolite levels in the Fenland study (B) of the isoleucine genetic score with and without the *GCKR* locus.
