# Supplementary material for: Genetic Predisposition to an Impaired Metabolism of the Branched-Chain Amino Acids and Risk of Type 2 Diabetes: A Mendelian Randomisation Analysis
Source: PLoS Med. 2016 Nov 29;13(11):e1002179. doi: 10.1371/journal.pmed.1002179 (PMC5127513; doi:10.1371/journal.pmed.1002179)
Supplement: S1 Table — (DOCX) [file pmed.1002179.s011.docx]

**S1 Table. Characteristics of the cohorts included in the genome-wide meta-analysis of branched chain amino acid levels.**

| **Variable** | **Fenland GWAS chip** | **Fenland UKBB chip** | **KORA** | **Twins UK** |
| --- | --- | --- | --- | --- |
| **Country** | United Kingdom | United Kingdom | Germany | United Kingdom |
| **Participants included in the GWAS, N** | 945 (945) | 8292 (8292) | 1768 (1763) | 6056 (5596) |
| **Age, mean years (SD)** | 46 (7) | 49 (7) | 61 (9) | 53 (14) |
| **Female sex, N (%)** | 543 (57) | 4364 (53) | 910 (51) | 5623 (93) |
| **Genotyping chip** | Affymetrix genome-Wide Human SNP Array 5.0 | Affymetrix UK Biobank Axiom Array | Affymetrix GeneChip array 6.0 | Illumina HumanHap300, HumanHap610Q, HumanHap1M and HumanHap1.2MDuo |
| **Imputation panel** | 1000 Genomes Phase 1 v3 | 1000 Genomes Phase 3 | HapMap 2 | HapMap 2 |
| **Metabolomics platform** | FIA-MS/MS and LC-MS/MS / AbsoluteIDQ® Biocrates p180 Kit | FIA-MS/MS and LC-MS/MS / AbsoluteIDQ® Biocrates p180 Kit | GC-MS and UPLC-MS/MS / Metabolon Inc. platform | GC-MS and UPLC-MS/MS / Metabolon Inc. platform |

Abbreviations: N, number of participants; SD, standard deviation; GWAS, genome-wide association study; FIA, flow injection analysis; MS/MS tandem mass spectrometry; LC, liquid chromatography; GC, gas chromatography; UPLC, ultra-performance liquid chromatography.
