# Supplementary material for: Genetic Predisposition to an Impaired Metabolism of the Branched-Chain Amino Acids and Risk of Type 2 Diabetes: A Mendelian Randomisation Analysis
Source: PLoS Med. 2016 Nov 29;13(11):e1002179. doi: 10.1371/journal.pmed.1002179 (PMC5127513; doi:10.1371/journal.pmed.1002179)
Supplement: S2 Table — (DOCX) [file pmed.1002179.s012.docx]

**S2 Table. Characteristics of the cohorts included in the analysis of the association of branched chain amino acid-raising alleles with type 2 diabetes.** Characteristics of the cohorts participating in DIAGRAM were reported by Morris and colleagues (Pubmed ID: 22885922). Details of the genotyping of rs1440581 in GoDARTS were reported by Menni and colleagues (Pubmed ID: 23884885).

| **Variable** | **EPIC-InterAct** | **UK Biobank** |
| --- | --- | --- |
| **Country** | Multiple European countries | United Kingdom |
| **Type 2 diabetes cases** | 6410 | 6627 |
| **Controls or Subcohort** | 8947 | 143766 |
| **Total** | 15357 | 150393 |
| **Age** | 53 (9) | 57 (8) |
| **Female sex, N (%)** | 9162 (60) | 79746 (53) |
| **Genotyping chip** | Illumina 660w quad and Illumina CoreExome chip | Affymetrix UK Biobank Axiom Array |
| **Imputation panel** | 1000 Genomes Phase 1 v3 | 1000 Genomes Phase 3 plus UK10K |

Abbreviations: N, number of participants.
