# Supplementary material for: Genetic Predisposition to an Impaired Metabolism of the Branched-Chain Amino Acids and Risk of Type 2 Diabetes: A Mendelian Randomisation Analysis
Source: PLoS Med. 2016 Nov 29;13(11):e1002179. doi: 10.1371/journal.pmed.1002179 (PMC5127513; doi:10.1371/journal.pmed.1002179)
Supplement: S3 Table — (DOCX) [file pmed.1002179.s013.docx]

**S3 Table. Characteristics of the studies included in the meta-analysis of the observational association between baseline levels of the branched chain amino acids and incident type 2 diabetes.**

| **Study** | **PMID** | **Design** | **Incident type 2 diabetes, N =** | **Controls, N =** | **Method for BCAA measurements** |
| --- | --- | --- | --- | --- | --- |
| 2011 Wang Nature Medicine - Framingham Offspring Study | 21423183 | Nested case-control | 189 | 189 | LC-MS/MS |
| 2011 Wang Nature Medicine - Malmö Diet and Cancer study | 21423183 | Nested case-control | 163 | 163 | LC-MS/MS |
| 2012 Wang-Sattler Molecular Systems Biology - KORA | 23010998 | Cohort | 91 | 785 | FIA-MS/MS and LC-MS/MS |
| 2013 Floegel Diabetes - EPIC Potsdam | 23043162 | Nested case-cohort | 800 | 2282 | FIA-MS/MS and LC-MS/MS |
| 2014 Palmer J Clin Endocrinol Metab - IRAS | 25423564 | Nested case-control | 76 | 70 | MS/MS |
| EPIC-Norfolk case-cohort study | This study | Nested case-cohort | 673 | 830 | UPLC-MS/MS |

Abbreviations: PMID, Pubmed ID of original article; N, number of participants; MS, mass spectrometry; MS/MS, tandem mass spectrometry; LC, liquid chromatography; UPLC, ultra-performance liquid chromatography; FIA, flow-injection analysis.
