# Supplementary material for: Genetic Predisposition to an Impaired Metabolism of the Branched-Chain Amino Acids and Risk of Type 2 Diabetes: A Mendelian Randomisation Analysis
Source: PLoS Med. 2016 Nov 29;13(11):e1002179. doi: 10.1371/journal.pmed.1002179 (PMC5127513; doi:10.1371/journal.pmed.1002179)
Supplement: S4 Table — (DOCX) [file pmed.1002179.s014.docx]

**S4 Table. Genetic scores for increased body mass index, insulin resistance and impaired insulin secretion.**

| **Phenotype** | **SNP** | **Chromosome** | **Position** | **Effect allele** | **Other allele** |
| --- | --- | --- | --- | --- | --- |
| **IR** | rs4846565 | 1 | 219722104 | G | A |
| **IR** | rs10195252 | 2 | 165513091 | T | C |
| **IR** | rs2943645 | 2 | 227099180 | T | C |
| **IR** | rs17036328 | 3 | 12390484 | T | C |
| **IR** | rs3822072 | 4 | 89741269 | A | G |
| **IR** | rs6822892 | 4 | 157734675 | A | G |
| **IR** | rs4865796 | 5 | 53272664 | A | G |
| **IR** | rs459193 | 5 | 55806751 | G | A |
| **IR** | rs2745353 | 6 | 127452935 | T | C |
| **IR** | rs731839 | 19 | 33899065 | G | A |
| **IS** | rs10946398 | 6 | 20661034 | C | A |
| **IS** | rs10830963 | 11 | 92708710 | G | C |
| **IS** | rs7903146 | 10 | 114758349 | T | C |
| **IS** | rs5015480 | 10 | 94465559 | C | T |
| **IS** | rs4502156 | 15 | 62383155 | T | C |
| **IS** | rs11603334 | 11 | 72432985 | G | A |
| **IS** | rs13266634 | 8 | 118184783 | C | T |
| **IS** | rs10811661 | 9 | 22134094 | T | C |
| **IS** | rs5219 | 11 | 17409572 | T | C |
| **IS** | rs7957197 | 12 | 121460686 | T | A |
| **IS** | rs174550 | 11 | 61571478 | T | C |
| **IS** | rs1800574 | 12 | 121416864 | T | C |
| **IS** | rs12686676 | 9 | 102632493 | G | A |
| **IS** | rs11605924 | 11 | 45873091 | A | C |
| **IS** | rs4607517 | 7 | 44235668 | A | G |
| **IS** | rs2237895 | 11 | 2857194 | C | A |
| **IS** | rs11672660 | 19 | 46180184 | T | C |
| **IS** | rs560887 | 2 | 169763148 | T | C |
| **IS** | rs12779790 | 10 | 12328010 | G | A |
| **IS** | rs933360 | 7 | 50758245 | T | C |
| **BMI** | rs10150332 | 14 | 79936964 | C | T |
| **BMI** | rs10767664 | 11 | 27725986 | A | T |
| **BMI** | rs10938397 | 4 | 45182527 | G | A |
| **BMI** | rs10968576 | 9 | 28414339 | G | A |
| **BMI** | rs11847697 | 14 | 30515112 | T | C |
| **BMI** | rs12444979 | 16 | 19933600 | C | T |
| **BMI** | rs13078807 | 3 | 85884150 | G | A |
| **BMI** | rs13107325 | 4 | 103188709 | T | C |
| **BMI** | rs1514175 | 1 | 74991644 | A | G |
| **BMI** | rs1555543 | 1 | 96944797 | C | A |
| **BMI** | rs1558902 | 16 | 53803574 | A | T |
| **BMI** | rs206936 | 6 | 34302869 | G | A |
| **BMI** | rs2112347 | 5 | 75015242 | T | G |
| **BMI** | rs2241423 | 15 | 68086838 | G | A |
| **BMI** | rs2287019 | 19 | 46202172 | C | T |
| **BMI** | rs2815752 | 1 | 72812440 | A | G |
| **BMI** | rs2867125 | 2 | 622827 | C | T |
| **BMI** | rs2890652 | 2 | 142959931 | C | T |
| **BMI** | rs29941 | 19 | 34309532 | G | A |
| **BMI** | rs3810291 | 19 | 47569003 | A | G |
| **BMI** | rs3817334 | 11 | 47650993 | T | C |
| **BMI** | rs4771122 | 13 | 28020180 | G | A |
| **BMI** | rs4836133 | 5 | 124332103 | A | C |
| **BMI** | rs4929949 | 11 | 8604593 | C | T |
| **BMI** | rs543874 | 1 | 177889480 | G | A |
| **BMI** | rs571312 | 18 | 57839769 | A | C |
| **BMI** | rs713586 | 2 | 25158008 | C | T |
| **BMI** | rs7138803 | 12 | 50247468 | A | G |
| **BMI** | rs7359397 | 16 | 28885659 | T | C |
| **BMI** | rs887912 | 2 | 59302877 | T | C |
| **BMI** | rs9816226 | 3 | 185834499 | T | A |
| **BMI** | rs987237 | 6 | 50803050 | G | A |

Abbreviations: IR, insulin resistance; IS, impaired insulin secretion; BMI, body mass index; SNP, single nucleotide polymorphism.
