# Supplementary material for: Genetic Predisposition to an Impaired Metabolism of the Branched-Chain Amino Acids and Risk of Type 2 Diabetes: A Mendelian Randomisation Analysis
Source: PLoS Med. 2016 Nov 29;13(11):e1002179. doi: 10.1371/journal.pmed.1002179 (PMC5127513; doi:10.1371/journal.pmed.1002179)
Supplement: S5 Table — (DOCX) [file pmed.1002179.s015.docx]

**S5 Table. Association of lead genetic variants at each locus with the levels of each of the branched chain amino acids.**

| **Locus** | **SNP** | **Lead SNP for** | **Genomic coordinates** | **Effect allele** | **Other allele** | **Metabolite** | **Beta per allele** | **SE** | **P-value** |
| --- | --- | --- | --- | --- | --- | --- | --- | --- | --- |
| ***PPM1K*** | rs7678928 | Isoleucine | 4:89222827 | t | c | Isoleucine | 0.090 | 0.0125 | 5.55E-19 |
|  |  |  |  |  |  | Leucine | 0.074 | 0.0133 | 2.50E-20 |
|  |  |  |  |  |  | Valine | 0.096 | 0.0134 | 3.45E-22 |
| ***GCKR*** | rs1260326 | Isoleucine | 2:27730940 | t | c | Isoleucine | 0.060 | 0.0123 | 1.14E-09 |
|  |  |  |  |  |  | Leucine | 0.049 | 0.0132 | 1.77E-07 |
|  |  |  |  |  |  | Valine | 0.050 | 0.0133 | 8.87E-07 |
| ***DDX19A*** | rs75950518 | Isoleucine | 16:70378917 | c | t | Isoleucine | 0.107 | 0.019 | 2.07E-08 |
|  |  |  |  |  |  | Leucine | 0.088 | 0.0203 | 1.56E-05 |
|  |  |  |  |  |  | Valine | 0.096 | 0.0206 | 2.83E-06 |
| ***TRMT61A*** | rs58101275 | Isoleucine | 14:104008420 | g | a | Isoleucine | 0.085 | 0.0153 | 2.78E-08 |
|  |  |  |  |  |  | Leucine | 0.051 | 0.0164 | 0.001721 |
|  |  |  |  |  |  | Valine | 0.055 | 0.0165 | 0.000912 |
| ***CBLN1*** | rs1420601 | Isoleucine | 16:49085649 | c | t | Isoleucine | 0.069 | 0.0125 | 3.71E-08 |
|  |  |  |  |  |  | Leucine | 0.046 | 0.0133 | 0.000525 |
|  |  |  |  |  |  | Valine | 0.055 | 0.0135 | 5.18E-05 |
| ***PPM1K*** | rs1440581 | Leucine, valine | 4:89226422 | c | t | Isoleucine | 0.086 | 0.0124 | 2.23E-18 |
|  |  |  |  |  |  | Leucine | 0.081 | 0.0132 | 3.86E-25 |
|  |  |  |  |  |  | Valine | 0.098 | 0.0133 | 4.40E-24 |

Abbreviations: SNP, single nucleotide polymorphism; N, number of participants; SE, standard error. Beta coefficients are in standardised units.
