# Supplementary material for: Genetic Predisposition to an Impaired Metabolism of the Branched-Chain Amino Acids and Risk of Type 2 Diabetes: A Mendelian Randomisation Analysis
Source: PLoS Med. 2016 Nov 29;13(11):e1002179. doi: 10.1371/journal.pmed.1002179 (PMC5127513; doi:10.1371/journal.pmed.1002179)
Supplement: S6 Table — (DOCX) [file pmed.1002179.s016.docx]

**S6 Table. Sensitivity analyses of the association of the isoleucine genetic score with type 2 diabetes.**

| **Exposure** | **Outcome** | **Model** | **Included loci** | **Number of SNPs** | **P-value** | **OR** | **95%CI of OR** |
| --- | --- | --- | --- | --- | --- | --- | --- |
| Isoleucine genetic score | Type 2 diabetes | Correlated  genetic variants | *PPM1K* | 9 | 2.1 x 10^-05^ | 1.43 | 1.21-1.68 |
| Isoleucine genetic score | Type 2 diabetes | Correlated  genetic variants | *PPM1K, DDX19A, CBLN1, TRMT61A* | 12 | 9.5 x 10^-08^ | 1.44 | 1.26-1.65 |
| Isoleucine genetic score | Type 2 diabetes | Independent  genetic variants | *PPM1K* | 1 | 0.0055 | 1.40 | 1.10-1.78 |
| Isoleucine genetic score | Type 2 diabetes | Independent  genetic variants | *PPM1K, DDX19A, CBLN1, TRMT61A* | 4 | 2.0 x 10^-05^ | 1.44 | 1.22-1.71 |
| Isoleucine genetic score | Type 2 diabetes | Independent  genetic variants | *DDX19A, CBLN1, TRMT61A* | 3 | 0.0011 | 1.48 | 1.17-1.88 |

Abbreviations: SNP, single nucleotide polymorphisms; SE, standard error; OR, odds ratio; CI, confidence interval.

ORs are reported per standard deviation of genetically-predicted change in isoleucine levels.
