# Supplementary material for: Genetic Predisposition to an Impaired Metabolism of the Branched-Chain Amino Acids and Risk of Type 2 Diabetes: A Mendelian Randomisation Analysis
Source: PLoS Med. 2016 Nov 29;13(11):e1002179. doi: 10.1371/journal.pmed.1002179 (PMC5127513; doi:10.1371/journal.pmed.1002179)
Supplement: S7 Table — The outcome of all the analyses was incident type 2 diabetes. (DOCX) [file pmed.1002179.s017.docx]

**S7 Table. Association of baseline isoleucine levels with incident type 2 diabetes by quartile of isoleucine levels.** The outcome of all the analyses was incident type 2 diabetes.

| **Study** | **Reference publication** | **Incident type 2 diabetes, N =** | **Controls, N =** | **Reference category** | **RR (95% CI) in quartile 2** | **RR (95% CI) in quartile 3** | **RR (95% CI) in quartile 4** |
| --- | --- | --- | --- | --- | --- | --- | --- |
| 2011 Wang Nature Medicine - Framingham Offspring Study | 21423183 | 189 | 189 | Quartile 1 | 1.11 (0.58-2.10) | 2.14 (1.07-4.27) | 3.14 (1.51-6.55) |
| 2011 Wang Nature Medicine - Malmö Diet and Cancer study | 21423183 | 163 | 163 | Quartile 1 | 1.02 (0.53-1.98) | 1.62 (0.77-3.42) | 2.37 (0.97-5.81) |
| EPIC-Norfolk case-cohort study | This study | 673 | 830 | Quartile 1 | 1.20 (0.76-1.89) | 1.31 (0.84-2.03) | 1.85 (1.19-2.89) |
| Meta-analysis | This study | 1025 | 1182 | Quartile 1 | 1.13 (0.82-1.56)* | 1.53 (1.10-2.13)** | 2.17 (1.53-3.08)*** |

Abbreviations: N, number of participants, RR, relative risk; CI, confidence interval. *I-squared = 0, P-value for heterogeneity = 0.92; **I-squared = 0, P-value for heterogeneity = 0.49; ***I-squared = 0, P-value for heterogeneity = 0.47.
