# Supplementary material for: Genetic Predisposition to an Impaired Metabolism of the Branched-Chain Amino Acids and Risk of Type 2 Diabetes: A Mendelian Randomisation Analysis
Source: PLoS Med. 2016 Nov 29;13(11):e1002179. doi: 10.1371/journal.pmed.1002179 (PMC5127513; doi:10.1371/journal.pmed.1002179)
Supplement: S8 Table — Only metabolites significantly associated after correction for multiple testing are reported. (DOCX) [file pmed.1002179.s018.docx]

**S8 Table. Association of the rs1440581 variant at the *PPM1K* locus with metabolites in the study by Shin et al. (Pubmed ID: 24816252).** Only metabolites significantly associated after correction for multiple testing are reported.

| **SNP** | **Effect allele** | **Other allele** | **Beta** | **SE** | **P-value** | **Metabolite** | **PMID** |
| --- | --- | --- | --- | --- | --- | --- | --- |
| rs1440581 | c | t | 0.0081 | 0.0015 | 1.14E-07 | isoleucine | 24816252 |
|  |  |  | 0.0081 | 0.0009 | 1.44E-19 | leucine | 24816252 |
|  |  |  | 0.0081 | 0.0012 | 2.56E-12 | valine | 24816252 |
|  |  |  | 0.014 | 0.0017 | 1.21E-16 | 3-methyl-2-oxovalerate | 24816252 |
|  |  |  | 0.0122 | 0.0021 | 6.55E-09 | 3-methyl-2-oxobutyrate | 24816252 |
|  |  |  | 0.0141 | 0.0022 | 2.43E-10 | 4-methyl-2-oxopentanoate | 24816252 |
|  |  |  | -0.0074 | 0.0017 | 2.43E-05 | 2-aminobutyrate | 24816252; 8069781 |
|  |  |  | 0.0166 | 0.0036 | 2.89E-06 | alpha-hydroxyisovalerate | 24816252 |

Abbreviations: SNP, single nucleotide polymorphism; SE, standard error; PMID, PubMed manuscript number. Beta coefficients are in log-10 transformed raw units.
