# Supplementary material for: Genetic Predisposition to an Impaired Metabolism of the Branched-Chain Amino Acids and Risk of Type 2 Diabetes: A Mendelian Randomisation Analysis
Source: PLoS Med. 2016 Nov 29;13(11):e1002179. doi: 10.1371/journal.pmed.1002179 (PMC5127513; doi:10.1371/journal.pmed.1002179)
Supplement: S9 Table — (DOCX) [file pmed.1002179.s019.docx]

**S9 Table. Association of branched chain amino acid-raising genetic variants with continuous metabolic traits in large-scale meta-analyses.**

| **SNP / Proxy** | **Locus** | **Effect / other allele** | **Phenotype** | **Source** | **PMID** | **Sample Size** | **Original units** | **Beta original units per allele** | **SE original units** | **Rescaling applied** | **Conversion factor** | **Beta standardised per allele** | **SE standardised** | **P-value** |
| --- | --- | --- | --- | --- | --- | --- | --- | --- | --- | --- | --- | --- | --- | --- |
| rs75950518 / rs12325419 | *DDX19A* | g/a | 2HG | MAGIC | 22885924 / 22581228 | 15234 | mmol/L | -0.011 | 0.028 | 1.716265 | 1.716265 | -0.00640927 | 0.0163145 | 0.6813 |
|  |  |  | BMI | GIANT | 25673413 | 236146 | Inverse normal transformed | 0.0043 | 0.0054 | assumed SD | 1 | 0.0043 | 0.0054 | 0.4259 |
|  |  |  | FPG | MAGIC | 22885924 / 22581228 | 58070 | mmol/L | -0.01 | 0.0048 | 0.6788359 | 0.6788359 | -0.0147311 | 0.00707093 | 0.03135 |
|  |  |  | HDL | GLGC | 24097068 | 92819 | SDs | 0.0176 | 0.0074 | none | 1 | 0.0176 | 0.0074 | 0.04229 |
|  |  |  | FI | MAGIC | 22885924 / 22581228 | 58070 | Ln-transformed picomoles | -0.013 | 0.0049 | 0.5931569 | 0.5931569 | -0.02191663 | 0.00826088 | 0.006891 |
|  |  |  | LDL | GLGC | 24097068 | 88432 | SDs | 0.0114 | 0.0081 | none | 1 | 0.0114 | 0.0081 | 0.2075 |
|  |  |  | TG | GLGC | 24097068 | 89483 | SDs of ln-transformed triglycerides | -0.0061 | 0.0071 | none | 1 | -0.0061 | 0.0071 | 0.1905 |
|  |  |  | WHRadjBMI | GIANT | 25673412 | 142745 | Inverse normal transformed BMI adjusted | -0.011 | 0.0063 | assumed SD | 1 | -0.011 | 0.0063 | 0.073 |
| rs1260326 / rs1260326 | *GCKR* | t/c | 2HG | MAGIC | 22885924 / 22581228 | 42854 | mmol/L | 0.059 | 0.011 | 1.716265 | 1.716265 | 0.03437698 | 0.00640927 | 1.45E-07 |
|  |  |  | BMI | GIANT | 25673413 | 328036 | Inverse normal transformed | -0.0126 | 0.0031 | assumed SD | 1 | -0.0126 | 0.0031 | 4.62E-05 |
|  |  |  | FPG | MAGIC | 22885924 / 22581228 | 133010 | mmol/L | 0.029 | 0.0021 | 0.6788359 | 0.6788359 | 0.04272019 | 0.00309353 | 2.17E-41 |
|  |  |  | HDL | GLGC | 24097068 | 187062 | SDs | -0.0113 | 0.0035 | none | 1 | -0.0113 | 0.0035 | 0.001737 |
|  |  |  | FI | MAGIC | 22885924 / 22581228 | 108557 | Ln-transformed picomoles | 0.019 | 0.0026 | 0.5931569 | 0.5931569 | 0.032032 | 0.00438333 | 3.84E-14 |
|  |  |  | LDL | GLGC | 24097068 | 172995 | SDs | 0.0206 | 0.0037 | none | 1 | 0.0206 | 0.0037 | 1.51E-07 |
|  |  |  | TG | GLGC | 24097068 | 177765 | SDs of ln-transformed triglycerides | 0.1148 | 0.0034 | none | 1 | 0.1148 | 0.0034 | 2.29E-239 |
|  |  |  | WHRadjBMI | GIANT | 25673412 | 210525 | Inverse normal transformed BMI adjusted | 0.01 | 0.0035 | assumed SD | 1 | 0.01 | 0.0035 | 0.0032 |
| rs1440581 / rs1440581 | *PPM1K* | c/t | 2HG | MAGIC | 22885924 / 22581228 | 15234 | mmol/L | -0.02 | 0.019 | 1.716265 | 1.716265 | -0.01165321 | 0.01107055 | 0.2843 |
|  |  |  | BMI | GIANT | 25673413 | 235533 | Inverse normal transformed | 0.0041 | 0.0036 | assumed SD | 1 | 0.0041 | 0.0036 | 0.2547 |
|  |  |  | FPG | MAGIC | 22885924 / 22581228 | 58070 | mmol/L | 0.0047 | 0.0031 | 0.6788359 | 0.6788359 | 0.00692362 | 0.00456664 | 0.1325 |
|  |  |  | HDL | GLGC | 24097068 | 94233 | SDs | -0.0081 | 0.0048 | none | 1 | -0.0081 | 0.0048 | 0.0702 |
|  |  |  | FI | MAGIC | 22885924 / 22581228 | 58070 | Ln-transformed picomoles | -0.0019 | 0.0032 | 0.5931569 | 0.5931569 | -0.0032032 | 0.00539486 | 0.5559 |
|  |  |  | LDL | GLGC | 24097068 | 89813 | SDs | 0.0077 | 0.0053 | none | 1 | 0.0077 | 0.0053 | 0.2826 |
|  |  |  | TG | GLGC | 24097068 | 90934 | SDs of ln-transformed triglycerides | 0.0035 | 0.0047 | none | 1 | 0.0035 | 0.0047 | 0.6961 |
|  |  |  | WHRadjBMI | GIANT | 25673412 | 142604 | Inverse normal transformed BMI adjusted | -0.0014 | 0.0043 | assumed SD | 1 | -0.0014 | 0.0043 | 0.74 |
| rs1420601 / rs1861569 | *CBLN1* | c/t | 2HG | MAGIC | 22885924 / 22581228 | 15234 | mmol/L | -0.014 | 0.019 | 1.716265 | 1.716265 | -0.00815725 | 0.01107055 | 0.4623 |
|  |  |  | BMI | GIANT | 25673413 | 236106 | Inverse normal transformed | 0.0177 | 0.0039 | assumed SD | 1 | 0.0177 | 0.0039 | 5.67E-06 |
|  |  |  | FPG | MAGIC | 22885924 / 22581228 | 58070 | mmol/L | -0.0002 | 0.0032 | 0.6788359 | 0.6788359 | -0.00029462 | 0.00471395 | 0.958 |
|  |  |  | HDL | GLGC | 24097068 | 92816 | SDs | -0.0072 | 0.0051 | none | 1 | -0.0072 | 0.0051 | 0.1885 |
|  |  |  | FI | MAGIC | 22885924 / 22581228 | 58070 | Ln-transformed picomoles | 0.0032 | 0.0032 | 0.5931569 | 0.5931569 | 0.00539486 | 0.00539486 | 0.3236 |
|  |  |  | LDL | GLGC | 24097068 | 88429 | SDs | 0.0017 | 0.0055 | none | 1 | 0.0017 | 0.0055 | 0.7711 |
|  |  |  | TG | GLGC | 24097068 | 89481 | SDs of ln-transformed triglycerides | 0.0053 | 0.0049 | none | 1 | 0.0053 | 0.0049 | 0.1968 |
|  |  |  | WHRadjBMI | GIANT | 25673412 | 142603 | Inverse normal transformed BMI adjusted | -0.0019 | 0.0045 | assumed SD | 1 | -0.0019 | 0.0045 | 0.67 |
| rs58101275 / rs58101275 | *TRMT61A* | g/a | 2HG | Fenland | This study | 9659 | SD | 0.0177688 | 0.0179 | none | 1 | 0.0177688 | 0.017911 | 0.321193 |
|  |  |  | BMI | Fenland | This study | 9796 | SD | 0.0252672 | 0.0177 | none | 1 | 0.0252672 | 0.0177955 | 0.15568 |
|  |  |  | FPG | Fenland | This study | 9787 | SD | 0.0276622 | 0.0170 | none | 1 | 0.0276622 | 0.0170798 | 0.105354 |
|  |  |  | HDL | Fenland | This study | 9802 | SD | 0.0023303 | 0.0160 | none | 1 | 0.0023303 | 0.0160298 | 0.88442 |
|  |  |  | FI | Fenland | This study | 8593 | SDs of ln-transformed insulin | 0.0341895 | 0.0187 | none | 1 | 0.0341895 | 0.0187629 | 0.068461 |
|  |  |  | LDL | Fenland | This study | 9727 | SD | -0.0131121 | 0.0174 | none | 1 | -0.0131121 | 0.0174453 | 0.452303 |
|  |  |  | TG | Fenland | This study | 9802 | SDs of ln-transformed triglycerides | 0.0173779 | 0.0170 | none | 1 | 0.0173779 | 0.017021 | 0.307296 |
|  |  |  | WHRadjBMI | Fenland | This study | 9780 | SD | -0.0009002 | 0.0112 | none | 1 | -0.0009002 | 0.0112453 | 0.936 |
| rs7678928 / rs7678928 | *PPM1K* | t/c | 2HG | MAGIC | 22885924 / 22581228 | 15234 | mmol/L | -0.038 | 0.019 | 1.716265 | 1.716265 | -0.0221411 | 0.01107055 | 0.04346 |
|  |  |  | BMI | GIANT | 25673413 | 235496 | Inverse normal transformed | 0.0037 | 0.0037 | assumed SD | 1 | 0.0037 | 0.0037 | 0.3173 |
|  |  |  | FPG | MAGIC | 22885924 / 22581228 | 58070 | mmol/L | 0.0003 | 0.0031 | 0.6788359 | 0.6788359 | 0.00044193 | 0.00456664 | 0.9276 |
|  |  |  | HDL | GLGC | 24097068 | 94288 | SDs | -0.0037 | 0.0048 | none | 1 | -0.0037 | 0.0048 | 0.4062 |
|  |  |  | FI | MAGIC | 22885924 / 22581228 | 58070 | Ln-transformed picomoles | -0.0006 | 0.0032 | 0.5931569 | 0.5931569 | -0.00101154 | 0.00539486 | 0.8433 |
|  |  |  | LDL | GLGC | 24097068 | 89865 | SDs | 0.0021 | 0.0053 | none | 1 | 0.0021 | 0.0053 | 0.8825 |
|  |  |  | TG | GLGC | 24097068 | 90990 | SDs of ln-transformed triglycerides | 0.0021 | 0.0047 | none | 1 | 0.0021 | 0.0047 | 0.9501 |
|  |  |  | WHRadjBMI | GIANT | 25673412 | 142468 | Inverse normal transformed BMI adjusted | -0.001 | 0.0044 | assumed SD | 1 | -0.001 | 0.0044 | 0.82 |
| Isoleucine genetic score | | | 2HG | MAGIC | 22885924 / 22581228 | 15234 | mmol/L | N/A | | | | -0.11002 | 0.076871 | 0.1523 |
|  |  |  | BMI | GIANT | 25673413 | 235496 | Inverse normal transformed |  |  |  |  | 0.096747 | 0.027637 | 0.0004* |
|  |  |  | FPG | MAGIC | 22885924 / 22581228 | 58070 | mmol/L |  |  |  |  | -0.02632 | 0.034312 | 0.4429 |
|  |  |  | HDL | GLGC | 24097068 | 94288 | SDs |  |  |  |  | 0.002124 | 0.036142 | 0.9531 |
|  |  |  | FI | MAGIC | 22885924 / 22581228 | 58070 | Ln-transformed picomoles |  |  |  |  | -0.02617 | 0.039999 | 0.5128 |
|  |  |  | LDL | GLGC | 24097068 | 89865 | SDs |  |  |  |  | 0.039795 | 0.039567 | 0.3145 |
|  |  |  | TG | GLGC | 24097068 | 90990 | SDs of ln-transformed triglycerides |  |  |  |  | 0.019599 | 0.035129 | 0.5769 |
|  |  |  | WHRadjBMI | GIANT | 25673412 | 142468 | Inverse normal transformed BMI adjusted |  |  |  |  | -0.04159 | 0.031763 | 0.1904 |

Abbreviations: SNP, single nucleotide polymorphism; SE, standard error; SD, standard deviation; PMID, PubMed manuscript number; 2HG, two hour glucose; BMI, body mass index; FPG, fasting plasma glucose; HDL, high-density lipoprotein cholesterol; FI, fasting insulin; LDL, low-density lipoprotein cholesterol; TG, triglycerides; WHRadjBMI, waist to hip ratio adjusted for body mass index.

Beta coefficients are in standardised units.

*P = 0.14 after the exclusion of *CBLN1* (see manuscript main text).
