# Supplementary material for: Genetic Predisposition to an Impaired Metabolism of the Branched-Chain Amino Acids and Risk of Type 2 Diabetes: A Mendelian Randomisation Analysis
Source: PLoS Med. 2016 Nov 29;13(11):e1002179. doi: 10.1371/journal.pmed.1002179 (PMC5127513; doi:10.1371/journal.pmed.1002179)
Supplement: S10 Table — (DOCX) [file pmed.1002179.s020.docx]

**S10 Table. Association of fasting insulin and body mass index with branched chain amino acid levels in the Fenland study.**

| **Exposure** | **Unit of exposure** | **Outcome** | **Sample size** | **Beta** | **Standard error** | **P-value** |
| --- | --- | --- | --- | --- | --- | --- |
| BMI | SD | Isoleucine | 9218 | 0.257 | 0.008 | 7.59E-214 |
| Ln-Insulin | SD | Isoleucine | 8028 | 0.262 | 0.009 | 5.21E-189 |
| BMI | SD | Leucine | 9218 | 0.191 | 0.009 | 7.21E-97 |
| Ln-Insulin | SD | Leucine | 8028 | 0.169 | 0.010 | 1.56E-65 |
| BMI | SD | Valine | 9218 | 0.280 | 0.009 | 7.78E-217 |
| Ln-Insulin | SD | Valine | 8028 | 0.282 | 0.010 | 1.28E-186 |

Beta coefficients are in standardised units of outcome per standardised unit of exposure.
