# Supplementary material for: Genetic Predisposition to an Impaired Metabolism of the Branched-Chain Amino Acids and Risk of Type 2 Diabetes: A Mendelian Randomisation Analysis
Source: PLoS Med. 2016 Nov 29;13(11):e1002179. doi: 10.1371/journal.pmed.1002179 (PMC5127513; doi:10.1371/journal.pmed.1002179)
Supplement: S11 Table — (DOCX) [file pmed.1002179.s021.docx]

**S11 Table. Association of body mass index, insulin resistance and impaired insulin secretion genetic scores with branched chain amino acids in the Fenland, Twins UK and KORA studies.**

| **Exposure** | **Outcome** | **Beta per allele** | **Standard error** | **Study** | **P-value** | **Heterogeneity P-value** | **I-square** |
| --- | --- | --- | --- | --- | --- | --- | --- |
| **IR score** | isoleucine | 0.015 | 0.003 | Meta-analysis | 0.000011 | 0.8115025 | 0 |
| **IS score** | isoleucine | 0.001 | 0.003 | Meta-analysis | 0.818655 | 0.6744607 | 0 |
| **BMI score** | isoleucine | 0.005 | 0.002 | Meta-analysis | 0.014781 | 0.3548243 | 0 |
| **IR score** | leucine | 0.013 | 0.003 | Meta-analysis | 0.000253 | 0.4126365 | 0 |
| **IS score** | leucine | -0.001 | 0.003 | Meta-analysis | 0.703919 | 0.7992458 | 0 |
| **BMI score** | leucine | 0.003 | 0.002 | Meta-analysis | 0.176709 | 0.8202943 | 0 |
| **IR score** | valine | 0.011 | 0.004 | Meta-analysis | 0.003678 | 0.8140155 | 0 |
| **IS score** | valine | 0.004 | 0.003 | Meta-analysis | 0.172492 | 0.7344247 | 0 |
| **BMI score** | valine | 0.008 | 0.002 | Meta-analysis | 0.001051 | 0.8613875 | 0 |
| **BMI score** | isoleucine | 0.006 | 0.003 | Fenland | 0.010923 | **N/A** | |
| **IR score** | isoleucine | 0.015 | 0.004 | Fenland | 0.000400 |  |  |
| **IS score** | isoleucine | 0.000 | 0.003 | Fenland | 0.974589 |  |  |
| **BMI score** | leucine | 0.003 | 0.003 | Fenland | 0.236036 |  |  |
| **IR score** | leucine | 0.015 | 0.004 | Fenland | 0.000792 |  |  |
| **IS score** | leucine | -0.002 | 0.003 | Fenland | 0.648435 |  |  |
| **BMI score** | valine | 0.007 | 0.003 | Fenland | 0.007339 |  |  |
| **IR score** | valine | 0.010 | 0.005 | Fenland | 0.023676 |  |  |
| **IS score** | valine | 0.005 | 0.003 | Fenland | 0.182594 |  |  |
| **IS score** | leucine | 0.000 | 0.004 | KORA + Twins UK | 0.972032 |  |  |
| **IS score** | isoleucine | 0.002 | 0.005 | KORA + Twins UK | 0.633025 |  |  |
| **IS score** | valine | 0.002 | 0.005 | KORA + Twins UK | 0.654842 |  |  |
| **IR score** | leucine | 0.009 | 0.005 | KORA + Twins UK | 0.094210 |  |  |
| **IR score** | valine | 0.012 | 0.007 | KORA + Twins UK | 0.066250 |  |  |
| **IR score** | isoleucine | 0.017 | 0.006 | KORA + Twins UK | 0.009138 |  |  |
| **BMI score** | isoleucine | 0.002 | 0.004 | KORA + Twins UK | 0.571162 |  |  |
| **BMI score** | leucine | 0.002 | 0.003 | KORA + Twins UK | 0.491803 |  |  |
| **BMI score** | valine | 0.008 | 0.004 | KORA + Twins UK | 0.058521 |  |  |
| **BMI score, adjusting for fasting insulin levels** | isoleucine | 0.002 | 0.003 | Fenland | 0.442000 |  |  |
| **BMI score, adjusting for fasting insulin levels** | leucine | 0.001 | 0.003 | Fenland | 0.811000 |  |  |
| **BMI score, adjusting for fasting insulin levels** | valine | 0.002 | 0.003 | Fenland | 0.393000 |  |  |
| **IR score, adjusting for BMI** | isoleucine | 0.016 | 0.004 | Fenland | 0.000064 |  |  |
| **IR score, adjusting for BMI** | leucine | 0.015 | 0.005 | Fenland | 0.001000 |  |  |
| **IR score, adjusting for BMI** | valine | 0.012 | 0.004 | Fenland | 0.009000 |  |  |

Abbreviations: IR, insulin resistance; IS, impaired insulin secretion; BMI, body mass index. Beta coefficients are in standardised units.
