# Supplementary material for: Genetic Predisposition to an Impaired Metabolism of the Branched-Chain Amino Acids and Risk of Type 2 Diabetes: A Mendelian Randomisation Analysis
Source: PLoS Med. 2016 Nov 29;13(11):e1002179. doi: 10.1371/journal.pmed.1002179 (PMC5127513; doi:10.1371/journal.pmed.1002179)
Supplement: S12 Table — (DOCX) [file pmed.1002179.s022.docx]

**S12 Table. Association of the insulin resistance genetic score with branched chain amino acids metabolites in the Twins UK and KORA studies.**

| **Exposure** | **Outcome** | **Beta per allele** | **Standard error** | **P-value** |
| --- | --- | --- | --- | --- |
| Insulin resistance genetic score | Leucine | 0.01 | 0.005 | 0.0942 |
|  | Isoleucine | 0.02 | 0.006 | 0.0091 |
|  | Valine | 0.01 | 0.007 | 0.0663 |
|  | Beta-hydroxyisovalerate | 0.01 | 0.006 | 0.2584 |
|  | 3-methyl-2-oxovalerate | 0.01 | 0.006 | 0.0112 |
|  | 3-methyl-2-oxobutyrate | 0.00 | 0.007 | 0.9068 |
|  | 4-methyl-2-oxopentanoate | 0.01 | 0.006 | 0.0377 |
|  | Propionylcarnitine | 0.01 | 0.005 | 0.0282 |
|  | Isobutyrylcarnitine | 0.00 | 0.006 | 0.7434 |
|  | Alpha-hydroxyisovalerate | 0.00 | 0.006 | 0.9823 |
|  | Isovalerylcarnitine | 0.01 | 0.006 | 0.1305 |

Beta coefficients are in standardised units.
