# Supplementary material for: Genetic Predisposition to an Impaired Metabolism of the Branched-Chain Amino Acids and Risk of Type 2 Diabetes: A Mendelian Randomisation Analysis
Source: PLoS Med. 2016 Nov 29;13(11):e1002179. doi: 10.1371/journal.pmed.1002179 (PMC5127513; doi:10.1371/journal.pmed.1002179)
Supplement: S13 Table — (DOCX) [file pmed.1002179.s023.docx]

**S13 Table. Levels of branched chain amino acids during the course of an oral glucose tolerance test in the SABRE study.**

| **Group** | **Metabolite** | **Timepoint during OGTT** | **Levels of amino acid mmol/L** | | | | |  |
| --- | --- | --- | --- | --- | --- | --- | --- | --- |
|  |  |  | **Minimum** | **25th percentile** | **Median** | **75th percentile** | **Maximum** | **P-value for difference between T120 and T0 during OGTT** |
| **Overall cohort** | Valine | 0 | 0.043 | 0.153 | 0.175 | 0.199 | 0.356 | <0.0001 |
|  |  | 120 | 0.024 | 0.145 | 0.168 | 0.193 | 0.38 |  |
|  | Isoleucine | 0 | 0.016 | 0.049 | 0.057 | 0.066 | 0.125 | <0.0001 |
|  |  | 120 | 0.00003 | 0.036 | 0.044 | 0.053 | 0.107 |  |
|  | Leucine | 0 | 0.026 | 0.079 | 0.09 | 0.104 | 0.275 | <0.0001 |
|  |  | 120 | 0.0013 | 0.063 | 0.075 | 0.091 | 0.214 |  |
| **Fasting insulin Q1** | Valine | 0 | 0.043 | 0.142 | 0.161 | 0.181 | 0.33 | N/A |
|  |  | 120 | 0.024 | 0.131 | 0.152 | 0.173 | 0.274 |  |
| **Fasting insulin Q2** | Valine | 0 | 0.064 | 0.151 | 0.171 | 0.192 | 0.33 |  |
|  |  | 120 | 0.025 | 0.143 | 0.163 | 0.182 | 0.321 |  |
| **Fasting insulin Q3** | Valine | 0 | 0.089 | 0.155 | 0.179 | 0.2 | 0.313 |  |
|  |  | 120 | 0.067 | 0.149 | 0.171 | 0.195 | 0.271 |  |
| **Fasting insulin Q4** | Valine | 0 | 0.073 | 0.169 | 0.191 | 0.216 | 0.356 |  |
|  |  | 120 | 0.063 | 0.166 | 0.189 | 0.214 | 0.38 |  |
| **Fasting insulin Q1** | Isoleucine | 0 | 0.016 | 0.043 | 0.05 | 0.059 | 0.099 |  |
|  |  | 120 | 0.00004 | 0.03 | 0.038 | 0.044 | 0.088 |  |
| **Fasting insulin Q2** | Isoleucine | 0 | 0.022 | 0.047 | 0.054 | 0.062 | 0.114 |  |
|  |  | 120 | 0.001 | 0.034 | 0.042 | 0.049 | 0.094 |  |
| **Fasting insulin Q3** | Isoleucine | 0 | 0.02 | 0.051 | 0.058 | 0.067 | 0.11 |  |
|  |  | 120 | 0.01 | 0.038 | 0.046 | 0.053 | 0.093 |  |
| **Fasting insulin Q4** | Isoleucine | 0 | 0.016 | 0.057 | 0.065 | 0.073 | 0.125 |  |
|  |  | 120 | 0.01 | 0.044 | 0.052 | 0.061 | 0.107 |  |
| **Fasting insulin Q1** | Leucine | 0 | 0.026 | 0.072 | 0.082 | 0.111 | 0.275 |  |
|  |  | 120 | 0.001 | 0.055 | 0.067 | 0.079 | 0.144 |  |
| **Fasting insulin Q2** | Leucine | 0 | 0.038 | 0.078 | 0.087 | 0.099 | 0.23 |  |
|  |  | 120 | 0.007 | 0.062 | 0.072 | 0.086 | 0.192 |  |
| **Fasting insulin Q3** | Leucine | 0 | 0.035 | 0.081 | 0.092 | 0.106 | 0.275 |  |
|  |  | 120 | 0.013 | 0.065 | 0.078 | 0.091 | 0.146 |  |
| **Fasting insulin Q4** | Leucine | 0 | 0.038 | 0.087 | 0.1 | 0.113 | 0.274 |  |
|  |  | 120 | 0.026 | 0.075 | 0.088 | 0.103 | 0.214 |  |
|  | | | | | | | | |
| **Overall cohort** | Valine | T120 - T0 difference | -0.1844 | -0.0241 | -0.0067 | 0.0098 | 0.1295 | N/A |
|  | Isoleucine | T120 - T0 difference | -0.07137 | -0.01953 | -0.01313 | -0.00643 | 0.03814 |  |
|  | Leucine | T120 - T0 difference | -0.21951 | -0.0265 | -0.0149 | -0.00433 | 0.07463 |  |
| **Fasting insulin Q1** | Valine | T120 - T0 difference | -0.1844 | -0.0255 | -0.0088 | 0.0059 | 0.09965 |  |
| **Fasting insulin Q2** | Valine | T120 - T0 difference | -0.12238 | -0.0235 | -0.0076 | 0.0089 | 0.1137 |  |
| **Fasting insulin Q3** | Valine | T120 - T0 difference | -0.1443 | -0.0251 | -0.0068 | 0.0101 | 0.1295 |  |
| **Fasting insulin Q4** | Valine | T120 - T0 difference | -0.1706 | -0.0209 | -0.0019 | 0.0167 | 0.12506 |  |
| **Fasting insulin Q1** | Isoleucine | T120 - T0 difference | -0.06591 | -0.0194 | -0.01295 | -0.00681 | 0.02353 |  |
| **Fasting insulin Q2** | Isoleucine | T120 - T0 difference | -0.056892 | -0.01938 | -0.01313 | -0.00641 | 0.03814 |  |
| **Fasting insulin Q3** | Isoleucine | T120 - T0 difference | -0.07137 | -0.02028 | -0.01364 | -0.00696 | 0.02969 |  |
| **Fasting insulin Q4** | Isoleucine | T120 - T0 difference | -0.05452 | -0.01894 | -0.01269 | -0.00487 | 0.03655 |  |
| **Fasting insulin Q1** | Leucine | T120 - T0 difference | -0.18307 | -0.02747 | -0.01576 | -0.0066 | 0.04803 |  |
| **Fasting insulin Q2** | Leucine | T120 - T0 difference | -0.1309 | -0.02503 | -0.01434 | -0.00492 | 0.07463 |  |
| **Fasting insulin Q3** | Leucine | T120 - T0 difference | -0.171 | -0.0292 | -0.01594 | -0.00461 | 0.06253 |  |
| **Fasting insulin Q4** | Leucine | T120 - T0 difference | -0.21951 | -0.025 | -0.013245 | -0.00049 | 0.0624 |  |

Abbreviations: Q, quartile; OGTT, oral glucose tolerance test.
