# Supplementary material for: Genetic Predisposition to an Impaired Metabolism of the Branched-Chain Amino Acids and Risk of Type 2 Diabetes: A Mendelian Randomisation Analysis
Source: PLoS Med. 2016 Nov 29;13(11):e1002179. doi: 10.1371/journal.pmed.1002179 (PMC5127513; doi:10.1371/journal.pmed.1002179)
Supplement: S14 Table — (DOCX) [file pmed.1002179.s024.docx]

**S14 Table. Association of fasting insulin with the response (i.e. difference of 120 minutes and 0 minutes) of levels of branched chain amino acids after a glucose challenge.**

| **Model** | **Outcome** | **Exposure** | **Adjustment** | **Beta coefficient per IU/L of fasting insulin** | **Standard error** | **P-value** |
| --- | --- | --- | --- | --- | --- | --- |
| **Continuous fasting insulin** | Change in Valine | Fasting insulin | none | 4.2x10^-4^ | 8.5x10^-5^ | 9.9x10^-7^ |
| **Continuous fasting insulin** | Change in Valine | Fasting insulin | age | 4.2x10^-4^ | 8.5x10^-5^ | 8.3x10^-7^ |
| **Continuous fasting insulin** | Change in Valine | Fasting insulin | age, sex | 3.9x10^-4^ | 8.5x10^-5^ | 6.3x10^-6^ |
| **Continuous fasting insulin** | Change in Valine | Fasting insulin | age, sex, ethnicity | 3.9x10^-4^ | 8.8x10^-5^ | 1.2x10^-5^ |
| **Continuous fasting insulin** | Change in Valine | Fasting insulin | age, sex, ethnicity, BMI | 3.3x10^-4^ | 1.6x10^-4^ | 4.4x10^-4^ |
| **Continuous fasting insulin** | Change in Valine | Fasting insulin | age, sex, ethnicity, BMI, fasting valine | 7.3x10^-4^ | 9.5x10^-5^ | 2.6x10^-17^ |
| **Fasting insulin quartiles** | Change in Valine | Fasting insulin Q2 | none | 0.0026 | 0.0017 | 0.13 |
| **Fasting insulin quartiles** | Change in Valine | Fasting insulin Q3 | none | 0.0033 | 0.046 | 0.046 |
| **Fasting insulin quartiles** | Change in Valine | Fasting insulin Q4 | none | 0.0087 | 0.0017 | 2.7x10^-7^ |
| **Fasting insulin quartiles** | Change in Valine | Fasting insulin Q2 | age | 0.0026 | 0.0017 | 0.12 |
| **Fasting insulin quartiles** | Change in Valine | Fasting insulin Q3 | age | 0.0034 | 0.0017 | 0.043 |
| **Fasting insulin quartiles** | Change in Valine | Fasting insulin Q4 | age | 0.0088 | 0.0017 | 2.2x10^-7^ |
| **Fasting insulin quartiles** | Change in Valine | Fasting insulin Q2 | age, sex | 0.0022 | 0.0017 | 0.2 |
| **Fasting insulin quartiles** | Change in Valine | Fasting insulin Q3 | age, sex | 0.0026 | 0.0017 | 0.12 |
| **Fasting insulin quartiles** | Change in Valine | Fasting insulin Q4 | age, sex | 0.0079 | 0.0017 | 3.9x10^-6^ |
| **Fasting insulin quartiles** | Change in Valine | Fasting insulin Q2 | age, sex, ethnicity | 0.0022 | 0.0017 | 0.2 |
| **Fasting insulin quartiles** | Change in Valine | Fasting insulin Q3 | age, sex, ethnicity | 0.0027 | 0.0017 | 0.12 |
| **Fasting insulin quartiles** | Change in Valine | Fasting insulin Q4 | age, sex, ethnicity | 0.008 | 0.0018 | 6.9x10^-6^ |
| **Fasting insulin quartiles** | Change in Valine | Fasting insulin Q2 | age, sex, ethnicity, BMI | 0.0018 | 0.0017 | 0.3 |
| **Fasting insulin quartiles** | Change in Valine | Fasting insulin Q3 | age, sex, ethnicity, BMI | 0.0021 | 0.0018 | 0.2 |
| **Fasting insulin quartiles** | Change in Valine | Fasting insulin Q4 | age, sex, ethnicity, BMI | 0.007 | 0.0019 | 2.9x10^-4^ |
| **Fasting insulin quartiles** | Change in Valine | Fasting insulin Q2 | age, sex, ethnicity, BMI, fasting valine | 0.0038 | 0.0015 | 0.013 |
| **Fasting insulin quartiles** | Change in Valine | Fasting insulin Q3 | age, sex, ethnicity, BMI, fasting valine | 0.0066 | 0.0016 | 3.9x10^-5^ |
| **Fasting insulin quartiles** | Change in Valine | Fasting insulin Q4 | age, sex, ethnicity, BMI, fasting valine | 0.0164 | 0.0018 | 1.5x10^-20^ |
| **Continuous fasting insulin** | Change in Isoleucine | Fasting insulin | none | 9.5x10^-5^ | 3x10^-5^ | 0.003 |
| **Continuous fasting insulin** | Change in Isoleucine | Fasting insulin | age | 9.6x10^-5^ | 3x10^-5^ | 0.003 |
| **Continuous fasting insulin** | Change in Isoleucine | Fasting insulin | age, sex | 9.5x10^-5^ | 3x10^-5^ | 0.003 |
| **Continuous fasting insulin** | Change in Isoleucine | Fasting insulin | age, sex, ethnicity | 1x10^-4^ | 3x10^-5^ | 0.001 |
| **Continuous fasting insulin** | Change in Isoleucine | Fasting insulin | age, sex, ethnicity, BMI | 1x10^-4^ | 4x10^-5^ | 0.001 |
| **Continuous fasting insulin** | Change in Isoleucine | Fasting insulin | age, sex, ethnicity, BMI, fasting isoleucine | 3x10^-4^ | 3x10^-5^ | 9.5x10^-23^ |
| **Fasting insulin quartiles** | Change in Isoleucine | Fasting insulin Q2 | none | 7x10^-4^ | 6x10^-4^ | 0.2 |
| **Fasting insulin quartiles** | Change in Isoleucine | Fasting insulin Q3 | none | -1x10^-4^ | 6x10^-4^ | 0.8 |
| **Fasting insulin quartiles** | Change in Isoleucine | Fasting insulin Q4 | none | 0.0015 | 6x10^-4^ | 0.016 |
| **Fasting insulin quartiles** | Change in Isoleucine | Fasting insulin Q2 | age | 7x10^-4^ | 6x10^-4^ | 0.2 |
| **Fasting insulin quartiles** | Change in Isoleucine | Fasting insulin Q3 | age | -1x10^-4^ | 6x10^-4^ | 0.8 |
| **Fasting insulin quartiles** | Change in Isoleucine | Fasting insulin Q4 | age | 0.0015 | 6x10^-4^ | 0.016 |
| **Fasting insulin quartiles** | Change in Isoleucine | Fasting insulin Q2 | age, sex | 7x10^-4^ | 6x10^-4^ | 0.3 |
| **Fasting insulin quartiles** | Change in Isoleucine | Fasting insulin Q3 | age, sex | -2 x10^-4^ | 6x10^-4^ | 0.8 |
| **Fasting insulin quartiles** | Change in Isoleucine | Fasting insulin Q4 | age, sex | 0.0015 | 6x10^-4^ | 0.019 |
| **Fasting insulin quartiles** | Change in Isoleucine | Fasting insulin Q2 | age, sex, ethnicity | 8x10^-4^ | 6x10^-4^ | 0.2 |
| **Fasting insulin quartiles** | Change in Isoleucine | Fasting insulin Q3 | age, sex, ethnicity | 2 x10^-6^ | 6x10^-4^ | 1 |
| **Fasting insulin quartiles** | Change in Isoleucine | Fasting insulin Q4 | age, sex, ethnicity | 0.0018 | 7x10^-4^ | 0.008 |
| **Fasting insulin quartiles** | Change in Isoleucine | Fasting insulin Q2 | age, sex, ethnicity, BMI | 9x10^-4^ | 6x10^-4^ | 0.2 |
| **Fasting insulin quartiles** | Change in Isoleucine | Fasting insulin Q3 | age, sex, ethnicity, BMI | 9x10^-5^ | 6x10^-4^ | 0.9 |
| **Fasting insulin quartiles** | Change in Isoleucine | Fasting insulin Q4 | age, sex, ethnicity, BMI | 0.0019 | 7x10^-4^ | 0.008 |
| **Fasting insulin quartiles** | Change in Isoleucine | Fasting insulin Q2 | age, sex, ethnicity, BMI, fasting isoleucine | 0.0017 | 6x10^-4^ | 0.002 |
| **Fasting insulin quartiles** | Change in Isoleucine | Fasting insulin Q3 | age, sex, ethnicity, BMI, fasting isoleucine | 0.0025 | 6x10^-4^ | 2x10^-5^ |
| **Fasting insulin quartiles** | Change in Isoleucine | Fasting insulin Q4 | age, sex, ethnicity, BMI, fasting isoleucine | 0.0065 | 6x10^-4^ | 9.5x10^-24^ |
| **Continuous fasting insulin** | Change in Leucine | Fasting insulin | none | 1.8x10^-4^ | 7x10^-5^ | 0.009 |
| **Continuous fasting insulin** | Change in Leucine | Fasting insulin | age | 1.8x10^-4^ | 7x10^-5^ | 0.01 |
| **Continuous fasting insulin** | Change in Leucine | Fasting insulin | age, sex | 1.9x10^-4^ | 7x10^-5^ | 0.006 |
| **Continuous fasting insulin** | Change in Leucine | Fasting insulin | age, sex, ethnicity | 1.7x10-4 | 7x10-5 | 0.015 |
| **Continuous fasting insulin** | Change in Leucine | Fasting insulin | age, sex, ethnicity, BMI | 1.7x10-4 | 8x10-5 | 0.027 |
| **Continuous fasting insulin** | Change in Leucine | Fasting insulin | age, sex, ethnicity, BMI, fasting leucine | 5.7x10-4 | 6x10-5 | 8.5x10-24 |
| **Fasting insulin quartiles** | Change in Leucine | Fasting insulin Q2 | none | 0.0019 | 0.0014 | 0.17 |
| **Fasting insulin quartiles** | Change in Leucine | Fasting insulin Q3 | none | -7.6x10^-4^ | 0.0013 | 0.6 |
| **Fasting insulin quartiles** | Change in Leucine | Fasting insulin Q4 | none | 0.0035 | 0.0014 | 0.01 |
| **Fasting insulin quartiles** | Change in Leucine | Fasting insulin Q2 | age | 0.0018 | 0.0014 | 0.17 |
| **Fasting insulin quartiles** | Change in Leucine | Fasting insulin Q3 | age | -7.7x10^-4^ | 0.0014 | 0.6 |
| **Fasting insulin quartiles** | Change in Leucine | Fasting insulin Q4 | age | 0.0034 | 0.0014 | 0.011 |
| **Fasting insulin quartiles** | Change in Leucine | Fasting insulin Q2 | age, sex | 0.002 | 0.0014 | 0.14 |
| **Fasting insulin quartiles** | Change in Leucine | Fasting insulin Q3 | age, sex | -5.0x10^-4^ | 0.0014 | 0.7 |
| **Fasting insulin quartiles** | Change in Leucine | Fasting insulin Q4 | age, sex | 0.0038 | 0.0014 | 0.006 |
| **Fasting insulin quartiles** | Change in Leucine | Fasting insulin Q2 | age, sex, ethnicity | 0.0019 | 0.0014 | 0.17 |
| **Fasting insulin quartiles** | Change in Leucine | Fasting insulin Q3 | age, sex, ethnicity | -7.5x10^-4^ | 0.0014 | 0.6 |
| **Fasting insulin quartiles** | Change in Leucine | Fasting insulin Q4 | age, sex, ethnicity | 0.0034 | 0.0014 | 0.018 |
| **Fasting insulin quartiles** | Change in Leucine | Fasting insulin Q2 | age, sex, ethnicity, BMI | 0.0018 | 0.0014 | 0.19 |
| **Fasting insulin quartiles** | Change in Leucine | Fasting insulin Q3 | age, sex, ethnicity, BMI | -8.7x10^-4^ | 0.0014 | 0.5 |
| **Fasting insulin quartiles** | Change in Leucine | Fasting insulin Q4 | age, sex, ethnicity, BMI | 0.0032 | 0.0016 | 0.041 |
| **Fasting insulin quartiles** | Change in Leucine | Fasting insulin Q2 | age, sex, ethnicity, BMI, fasting leucine | 0.0033 | 0.001 | 0.001 |
| **Fasting insulin quartiles** | Change in Leucine | Fasting insulin Q3 | age, sex, ethnicity, BMI, fasting leucine | 0.0042 | 0.001 | 9.4x10^-4^ |
| **Fasting insulin quartiles** | Change in Leucine | Fasting insulin Q4 | age, sex, ethnicity, BMI, fasting leucine | 0.012 | 0.0011 | 5.3x10^-5^ |

Abbreviations: Q, quartile; BMI, body mass index. Beta coefficients are in mmol/L of amino acid levels.
