# Supplementary material for: Genetic Predisposition to an Impaired Metabolism of the Branched-Chain Amino Acids and Risk of Type 2 Diabetes: A Mendelian Randomisation Analysis
Source: PLoS Med. 2016 Nov 29;13(11):e1002179. doi: 10.1371/journal.pmed.1002179 (PMC5127513; doi:10.1371/journal.pmed.1002179)
Supplement: S15 Table — The table reports the association of BCAA genetic scores with all measured BCAA-related metabolites in the EPIC-Norfolk, TwinsUK, and KORA studies. The table also reports the association of BCAA-related metabolites with incident type 2 diabetes in the EPIC-Norfolk case-cohort study. (DOCX) [file pmed.1002179.s025.docx]

**S15 Table. Branched chain amino acid pathway analysis.** The Table reports the association of branched chain amino acid **(**BCAA) genetic scores with all measured BCAA-related metabolites in the EPIC-Norfolk, Twins UK and KORA studies. The Table also reports the association of BCAA-related metabolites with incident type 2 diabetes in the EPIC-Norfolk case-cohort study.

| **Analysis** | **Exposure** | **Outcome** | **Position in the BCAA pathway** | **Sample size** | **Hazard ratio** | **Lower bound of 95% CI of the HR** | **Upper bound of 95% CI of the HR** | **Beta per allele** | **Standard error** | **P-value** |
| --- | --- | --- | --- | --- | --- | --- | --- | --- | --- | --- |
| **Genetic score to metabolite levels** | Leucine or valine genetic scores | Isoleucine | upsteam | 8693 | N/A | | | 0.091317 | 0.02 | 5.98E-08 |
|  |  | Leucine | upsteam | 8693 |  |  |  | 0.108334 | 0.01 | 8.32E-19 |
|  |  | Valine | upsteam | 8691 |  |  |  | 0.117364 | 0.02 | 4.64E-12 |
|  |  | 3-methyl-2-oxovalerate | upsteam | 8691 |  |  |  | 0.12505 | 0.01 | 1.49E-19 |
|  |  | 3-methyl-2-oxobutyrate | upsteam | 8691 |  |  |  | 0.131626 | 0.02 | 3.30E-14 |
|  |  | 3-hydroxy-2-ethylpropionate | not classified | 8688 |  |  |  | 0.118278 | 0.02 | 2.44E-13 |
|  |  | 4-methyl-2-oxopentanoate | upsteam | 1332 |  |  |  | 0.128033 | 0.036024 | 0.000393 |
|  |  | 2-hydroxy-3-methylvalerate | upstream | 1324 |  |  |  | 0.107158 | 0.03745 | 0.004285 |
|  |  | Ethylmalonate | not classified | 1331 |  |  |  | -0.058 | 0.037735 | 0.124496 |
|  |  | 3-hydroxyisobutyrate | downstream | 1332 |  |  |  | 0.014565 | 0.038431 | 0.704762 |
|  |  | Beta-hydroxyisovalerate | not classified | 8691 |  |  |  | -0.00351 | 0.015601 | 0.822115 |
|  |  | Alpha-hydroxyisovalerate | upstream | 8691 |  |  |  | 0.094532 | 0.016431 | 8.76E-09 |
|  |  | Alpha-hydroxyisocaproate | upstream | 1330 |  |  |  | 0.123133 | 0.036097 | 0.000666 |
|  |  | Methylsuccinate | not classified | 1332 |  |  |  | -0.05297 | 0.038528 | 0.169398 |
|  |  | N-acetylvaline | not classified | 1329 |  |  |  | 0.10032 | 0.03798 | 0.008354 |
|  |  | Propionylcarnitine | downstream | 8691 |  |  |  | -0.04381 | 0.02 | 0.00995 |
|  |  | Isovalerylcarnitine | downstream | 8684 |  |  |  | -0.01398 | 0.02 | 0.420023 |
|  |  | Isobutyrylcarnitine | downstream | 8659 |  |  |  | 0.02459 | 0.02 | 0.134604 |
| **Genetic score to metabolite levels** | Isoleucine genetic score | Isoleucine | upsteam | 8693 |  |  |  | 0.055022 | 0.009055 | 1.23E-09 |
|  |  | Leucine | upsteam | 8693 |  |  |  | 0.051155 | 0.006784 | 4.67E-14 |
|  |  | Valine | upsteam | 8691 |  |  |  | 0.057521 | 0.009398 | 9.32E-10 |
|  |  | 3-methyl-2-oxovalerate | upsteam | 8691 |  |  |  | 0.073446 | 0.008128 | 1.62E-19 |
|  |  | 3-methyl-2-oxobutyrate | upsteam | 8691 |  |  |  | 0.063755 | 0.009583 | 2.87E-11 |
|  |  | 3-hydroxy-2-ethylpropionate | not classified | 8688 |  |  |  | 0.062139 | 0.008884 | 2.66E-12 |
|  |  | 4-methyl-2-oxopentanoate | upsteam | 1332 |  |  |  | 0.054653 | 0.020859 | 0.008889 |
|  |  | 2-hydroxy-3-methylvalerate | upstream | 1324 |  |  |  | 0.034419 | 0.021642 | 0.111982 |
|  |  | Ethylmalonate | not classified | 1331 |  |  |  | -0.02833 | 0.021808 | 0.194126 |
|  |  | 3-hydroxyisobutyrate | downstream | 1332 |  |  |  | 0.025038 | 0.022211 | 0.259821 |
|  |  | Beta-hydroxyisovalerate | not classified | 8691 |  |  |  | 0.011189 | 0.008685 | 0.197654 |
|  |  | Alpha-hydroxyisovalerate | upstream | 8691 |  |  |  | 0.048672 | 0.008991 | 6.18E-08 |
|  |  | Alpha-hydroxyisocaproate | upstream | 1330 |  |  |  | 0.063676 | 0.020874 | 0.00233 |
|  |  | Methylsuccinate | not classified | 1332 |  |  |  | -0.00957 | 0.022241 | 0.667141 |
|  |  | N-acetylvaline | not classified | 1329 |  |  |  | 0.063176 | 0.021958 | 0.004078 |
|  |  | Propionylcarnitine | downstream | 8691 |  |  |  | 0.016763 | 0.008344 | 0.044529 |
|  |  | Isovalerylcarnitine | downstream | 8684 |  |  |  | 0.005993 | 0.008933 | 0.502284 |
|  |  | Isobutyrylcarnitine | downstream | 8659 |  |  |  | 0.00807 | 0.00907 | 0.373603 |
| **Metabolite levels to incident T2D** | Isoleucine | Incident type 2 diabetes | upsteam | 1466 | 1.31 | 1.11 | 1.54 | 0.268354 | 0.08294 | 0.001214 |
|  | Leucine |  | upsteam | 1466 | 1.22 | 1.03 | 1.43 | 0.19725 | 0.083456 | 0.018102 |
|  | Valine |  | upsteam | 1464 | 1.34 | 1.12 | 1.61 | 0.295459 | 0.091353 | 0.00122 |
|  | 3-methyl-2-oxovalerate |  | upsteam | 1464 | 1.62 | 1.35 | 1.95 | 0.484345 | 0.094654 | 3.10E-07 |
|  | 3-methyl-2-oxobutyrate |  | upsteam | 1464 | 1.60 | 1.33 | 1.92 | 0.467558 | 0.093527 | 5.76E-07 |
|  | 3-hydroxy-2-ethylpropionate |  | not classified | 1460 | 1.40 | 1.21 | 1.63 | 0.337906 | 0.077163 | 1.19E-05 |
|  | 4-methyl-2-oxopentanoate |  | upsteam | 1464 | 1.47 | 1.23 | 1.76 | 0.385561 | 0.092137 | 2.86E-05 |
|  | 2-hydroxy-3-methylvalerate |  | upstream | 1456 | 1.36 | 1.18 | 1.58 | 0.310773 | 0.075029 | 3.44E-05 |
|  | Ethylmalonate |  | not classified | 1463 | 1.28 | 1.13 | 1.45 | 0.246273 | 0.063519 | 0.000106 |
|  | 3-hydroxyisobutyrate |  | downstream | 1464 | 1.32 | 1.13 | 1.54 | 0.277088 | 0.079252 | 0.000472 |
|  | Beta-hydroxyisovalerate |  | not classified | 1464 | 1.24 | 1.06 | 1.45 | 0.218188 | 0.079789 | 0.006247 |
|  | Alpha-hydroxyisovalerate |  | upstream | 1464 | 1.22 | 1.05 | 1.41 | 0.19797 | 0.075604 | 0.008831 |
|  | Alpha-hydroxyisocaproate |  | upstream | 1461 | 1.22 | 1.03 | 1.45 | 0.200006 | 0.08905 | 0.024704 |
|  | Methylsuccinate |  | not classified | 1463 | 1.18 | 1.02 | 1.36 | 0.163085 | 0.073018 | 0.025517 |
|  | N-acetylvaline |  | not classified | 1461 | 1.19 | 1.02 | 1.39 | 0.173294 | 0.079259 | 0.028785 |
|  | Propionylcarnitine |  | downstream | 1464 | 1.15 | 0.98 | 1.34 | 0.139541 | 0.079178 | 0.078007 |
|  | Isovalerylcarnitine |  | downstream | 1457 | 1.13 | 0.97 | 1.32 | 0.123075 | 0.077552 | 0.112511 |
|  | Isobutyrylcarnitine |  | downstream | 1430 | 0.82 | 0.70 | 0.96 | -0.19692 | 0.080028 | 0.013868 |

Abbreviations: T2D, type 2 diabetes; HR, hazard ratio; CI, confidence interval; BCAA, branched chain amino acids. Beta coefficients are in standardised units. Hazard ratios are per 1 standard deviation change in baseline metabolite levels.
