# Supplementary material for: Genetic Predisposition to an Impaired Metabolism of the Branched-Chain Amino Acids and Risk of Type 2 Diabetes: A Mendelian Randomisation Analysis
Source: PLoS Med. 2016 Nov 29;13(11):e1002179. doi: 10.1371/journal.pmed.1002179 (PMC5127513; doi:10.1371/journal.pmed.1002179)
Supplement: S1 Text — (DOCX) [file pmed.1002179.s026.docx]

**S1 Text - Supplementary Methods.**

1. **Genome-wide association studies (GWAS) of isoleucine, leucine and valine plasma levels**

**1.1 Fenland study analyses**

*The Fenland study*

The Fenland study is a population-based cohort study of 12,435 non-diabetic participants born between 1950 and 1975 (aged 30-62 years at baseline). Participants were recruited from general practice surgeries in Cambridge, Ely or Wisbech (Cambridgeshire, United Kingdom) and underwent detailed metabolic phenotyping, including genome-wide genotyping and targeted metabolomics profiling in subsets of the cohort (URL:http://www.mrc-epid.cam.ac.uk/research/studies/fenland). This study focused on 9,237 individuals with available genome-wide genotyping and metabolomics profiling.

*Metabolite measurements*

The levels of 175 metabolites were measured in the Fenland study by the AbsoluteIDQ® Biocrates p180 Kit (Biocrates Life Sciences AG, Innsbruck, Austria) as reported elsewhere in detail [[1](#_ENREF_1),[2](#_ENREF_2)]. We used a Waters Acquity ultra-performance liquid chromatography (UPLC; Waters ltd, Manchester, UK) system coupled to an ABSciex 5500 Qtrap mass spectrometer (Sciex ltd, Warrington, UK). Samples were derivatised and extracted using a Hamilton STAR liquid handling station (Hamilton Robotics Ltd, Birmingham, UK). Flow injection analysis coupled with tandem mass spectrometry (FIA-MS/MS) using multiple reaction monitoring (MRM) in positive mode ionisation was performed to measure the relative levels of acylcarnitines, phosphatidylcholines, lysophosphatidylcholines and sphingolipids. The level of hexose was measured in negative ionisation mode. Ultra-performance liquid chromatography coupled with tandem mass spectrometry (UPLC-MS/MS) using MRM was performed to measure the concentration of amino acids and biogenic amines. The chromatography consisted of a 5 minute gradient starting at 100% aqueous (0.2% Formic acid) increasing to 95% acetonitrile (0.2% Formic acid) over a Waters Acquity UPLC BEH C18 column (2.1 x 50 mm, 1.7 μm, with guard column). Isotopically labelled internal standards are integrated within the Biocrates p180 Kit for quantification. Data was processed in the Biocrates Met*IDQ* software. Raw metabolite readings underwent extensive quality control procedures. Firstly, we excluded from any further analysis metabolites for which the number of measurements below the limit of quantitification (LOQ) exceeded 5% of measured samples. Excluded metabolites were carnosine, dopamine, putrescine, asymmetric dimethyl arginine, dihydroxyphenylalanine, nitrotyrosine, spermine, sphingomyelines SM(22:3), SM(26:0), SM(26:1), SM(24:1-OH), phosphatidylcholine acyl-alky 44:4, and phosphatidylcholine diacyl C30:2. Secondly, in samples with detectable but not quantifiable peaks, we assigned random values between 0 and the run-specific LOQ of a given metabolite. Finally, we corrected for batch-effects with a “location-scale” approach, i.e. with normalization for mean and standard deviation of batches [[3](#_ENREF_3)].

*Genome-wide genotyping*

In the Fenland study, genome wide genotyping was performed in two waves. In the first, 1,402 participants were genotyped using the Affymetrix Genome-Wide Human SNP Array 5.0, with imputation to the 1000 Genomes Phase 1 v3 reference panel (URL: http://www.1000genomes.org/). A total of 945 out of those 1,402 participants had available genome-wide genotyping and metabolomics and contributed to this study (**S1 Table**). In the second, 9,369 participants were genotyped using the Affymetrix UK Biobank Axiom Array, with imputation to the 1000 Genomes phase 3 panel (http://www.1000genomes.org/). A total of 8,292 out of those 9,369 participants had available genome-wide genotyping and metabolomics and contributed to this study (**S1 Table**). Imputation was performed using the IMPUTE v2 software.[[4](#_ENREF_4)]

*GWAS analysis in Fenland*

Before analysis, metabolite levels were log10-transformed, winsorised to 5 standard deviations (SDs) and standardised to a mean of 0 and a SD of 1. GWAS analysis was performed separately in the two Fenland study subsets using SNPTEST v2, using linear regression models and assuming additive effects [[5](#_ENREF_5)]. Analyses were adjusted for age, sex and the first four principal components. As part of quality control procedures, we dropped variants for which the standard error could not be modelled or exceeded 10, the p-value for violations of Hardy-Weimberg equilibrium was below 10^-6^, the absolute value of the beta coefficient exceeded 5 or the minor allele frequency was below 1%. The results of the GWAS analyses in the two subsets were meta-analysed using METAL [[6](#_ENREF_6)]. We performed a meta-analysis of beta coefficients and standard errors with genomic control correction.

**1.2 Publicly-available GWAS results of isoleucine, leucine and valine**

Publicly-available GWAS results from the study of Shin et al. [[7](#_ENREF_7)] were downloaded from the Metabolomics GWAS sever (http://mips.helmholtz-muenchen.de/proj/GWAS/ gwas/index.php?task=download). Shin et al. reported the results of GWAS studies of log10-transformed metabolite levels in the KORA and Twins UK studies [[7](#_ENREF_7)]. For that study, metabolites were measured using an untargeted metabolomics platform (gas-chromatography mass spectrometry or ultra-performance liquid chromatography coupled with tandem mass spectrometry [GC-MS or UPLC-MS/MS] by Metabolon Inc.). Genome-wide analysis was conducted as described in Shin et al. [[7](#_ENREF_7)], with imputation to the HapMap 2 reference panel (URL: http://hapmap.ncbi.nlm.nih.gov/).

**1.3 GWAS meta-analysis**

GWAS results for isoleucine, leucine and valine from the effort of Shin et al. were meta-analysed with results from the Fenland study. We performed a meta-analysis of Z-scores using METAL [[6](#_ENREF_6)]. We applied a filter for variants with a minor allele frequency below 1% and genomic control correction. We used the conventional genome-wide significance p-value threshold of 5 x 10^-8^ to define loci associated with the levels of a given metabolite. A genomic locus were defined as the 1 Mb region around the lead single nucleotide polymorphism. Regional plots were drawn using the LocusZoom software [[8](#_ENREF_8)]. Manhattan plots were drawn using the R package EasyStrata v8.6 (URL: www.genepiregensburg.de/easystrata).

**2.** **Association of branched chain amino acid (BCAA)-raising alleles with type 2 diabetes**

*The EPIC-InterAct study*

EPIC-InterAct is a case-cohort study nested within the European Prospective Investigation into Cancer and Nutrition (EPIC) study, a cohort study of 340,234 European participants. Participants of EPIC were followed-up for an average of 8 years, providing 4 million person-years of follow-up [[9](#_ENREF_9)]. During this time, a total of 12,403 individuals who were free of diabetes at baseline were identified as having developed type 2 diabetes and constitute the incident disease group of InterAct [[9](#_ENREF_9)]. EPIC-InterAct has also defined a randomly-selected subcohort of 16,154 controls who were free of diabetes at baseline [[9](#_ENREF_9)]. Genotyping in the InterAct study was performed in a total of 16,691 samples using the Illumina 660w quad (N=9,294) and Illumina CoreExome chip (N=7,397). The EPIC-Norfolk case-cohort study (N=1,503, see description below) is part of InterAct and has also contributed available GWAS data (N=1,334) to the DIAbetes Genetics Replication And Meta-analysis (DIAGRAM, see below). Therefore, to avoid overlap with DIAGRAM participants, the contribution of InterAct to the type 2 diabetes genetic association analysis of this study amounts to 6,410 incident cases of type 2 diabetes and 8,947 non-cases with available genotype data after the exclusion of EPIC-Norfolk participants. A GWAS analysis for type 2 diabetes was performed in EPIC-InterAct using SNPTEST v2 [[5](#_ENREF_5)]. InterAct GWAS results were meta-analysed with DIAGRAM results using METAL [[6](#_ENREF_6)].

*DIAGRAM*

The DIAbetes Genetics Replication And Meta-analysis is a consortium of research groups with an interest in the genetic basis of type 2 diabetes [[10](#_ENREF_10)]. DIAGRAM published the largest meta-analysis of GWAS for type 2 diabetes in individuals of European descent. Summary results were made publicly available for a meta-analysis of 2.5 million SNPs in up to 12,171 cases and 56,862 controls, with imputation to the HapMap 2 reference panel [[10](#_ENREF_10)]. Summary results for a subset of ~130,000 SNPs, genotyped with the Metabochip array, in an additional 22,669 cases and 58,119 controls are also available [[10](#_ENREF_10)]. For this study, we used the DIAGRAM Metabochip results (where available) and the GWAS results (where Metabochip was not available).

*GoDARTS*

The Genetics of Diabetes and Audit Research Tayside Study is a type 2 diabetes case-control study [[11](#_ENREF_11)]. In this study, we meta-analysed with InterAct and UK Biobank the combined estimate of the association of rs1440581 at the *PPM1K* locus with type 2 diabetes in GoDARTS plus DIAGRAM (see above), including 4,961 cases and 5,948 controls from GoDARTS. This was published by Menni et al. as part of an evaluation of the role of 3-methyl-2-oxovalerate in diabetes [[12](#_ENREF_12)].

*UK Biobank*

The UK Biobank study is a population-based cohort study of ~500,000 people aged between 40-69 years who were recruited in 2006-2010 from several centres across the United Kingdom [[13](#_ENREF_13)]. In this study, we assessed the association of BCAA-associated SNPs with prevalent type 2 diabetes in 150,393 individuals (6,627 cases and 143,766 controls) of the initial UK Biobank dataset (URL: http://www.ukbiobank.ac.uk/). Type 2 diabetes was defined on the basis of self-reported physician diagnosis at nurse interview or digital questionnaire, age at diagnosis > 36 years, and use of oral anti-diabetic medications. InterAct plus DIAGRAM association results were meta-analysed with UK Biobank results using fixed effect models using the STATA software.

**3.** **Mendelian randomisation analysis**

We estimated the association between a genetically-predicted difference of 1 standard deviation (SD) in isoleucine, leucine or valine plasma levels and type 2 diabetes risk. For each of the BCAAs, we constructed weighted genetic risk scores including (a) the lead SNP (*analysis of independent genetic variants*) or (b) genome-wide significant SNPs in imperfect linkage disequilibrium (r^2^ < 0.8 for all pairwise SNPs comparisons; *analysis of correlated genetic variants*) at each locus identified by the genome-wide meta-analysis for that metabolite. Weights were derived from the Fenland study genetic analysis and used to scale the effect of each genetic score to 1 SD. In order to allow comparability between Mendelian randomisation estimates reported in this study and observational estimates reported in the literature, SNP weights were derived to reflect an effect in SD units of untransformed metabolite levels.

Lead SNPs (nearest gene) included in the genetic scores used in independent variants analysis were reported in the main text. Analyses of correlated genetic variants modelled the estimates of 12 SNPs at 4 loci (i.e. rs7678928, rs1440581, rs10024717, rs881561, rs9637599, rs893970, rs17732955, rs28884607, rs1808859 near *PPM1K*, rs75950518 near *DDX19A*, rs58101275 near *TRMT61*, and rs1420601 near *CBLN1*) for isoleucine; 7 SNPs (rs1440581, rs7678928, rs10024717, rs2869926, rs9637599, rs893970, rs28789746) at the *PPM1K* locus for leucine; and 8 SNPs (rs1440581, rs7678928, rs9637599, rs10024717, rs2869926, rs893970, rs17732955, rs1808859) at the *PPM1K* locus for valine. For the analysis of correlated genetic variants, SNPs were ranked by p-value for association with BCAA levels and were iteratively selected for inclusion in the model with the following criteria: (a) association at genome-wide level of statistical significance (P < 5 x 10^-8^) with the levels of a given BCAA; (b) pairwise correlation with other SNPs included in the model < 0.8 for each comparison. Correlations were derived from the 1000 Genomes CEU population using SNAP [[14](#_ENREF_14)].

We used estimates of “SNP to metabolite” and “SNP to type 2 diabetes” associations to calculate genetically-predicted estimates of each “metabolite to type 2 diabetes” association [[15](#_ENREF_15)]. Estimates of multiple SNPs contributing to a given genetic score were pooled using an inverse-variance weighted method [[15](#_ENREF_15)]. For the analysis of correlated genetic variants, estimates were pooled with a method that accounts for the correlation between genetic variants [[16](#_ENREF_16)].

**4.** **Systematic review and meta-analysis of the prospective association between BCAA levels and incident type 2 diabetes**

*Systematic review of the literature*

We conducted a systematic review of the literature about the prospective association of BCAA levels with incident type 2 diabetes in observational studies (**S2 Text**). We conducted a MEDLINE search updated to the 5^th^ of November 2015 with the following search strategy: *(branched chain amino acid OR branched chain amino acids OR BCAA OR BCAAs OR isoleucine OR leucine OR valine OR metabolomics) AND (diabetes OR “type 2 diabetes”) AND (cohort OR prospective OR longitudinal OR incidence OR incident) AND English[Language]*. Titles, abstracts and full articles were screened with the goal of identifying observational studies with a prospective design (i.e. cohort, nested case-control or nested case-cohort), which investigated the baseline levels of plasma BCAAs in adult diabetes-free individuals and related these baseline levels to the risk of incident type 2 diabetes (**S4 Fig**).

*Meta-analysis of prospective studies of BCAA levels and risk of incident type 2 diabetes*

Data on the relative risk of type 2 diabetes per 1 SD difference in isoleucine, leucine or valine was extracted from five prospective studies. Results were meta-analysed with new data from the EPIC-Norfolk type 2 diabetes case-cohort study (see below). The meta-analysis was conducted using fixed effect models. We also meta-analysed associations with type 2 diabetes in quartiles of isoleucine levels from the EPIC-Norfolk case-cohort study, the Framingham Offspring study, and the Malmö Diet and Cancer study using fixed effect models. The I-squared statistic was used to quantify heterogeneity.

*EPIC-Norfolk analyses contributing to the meta-analysis*

Levels of BCAAs were winsorised to 5 SDs and standardised to a mean of 0 and a standard deviation of 1 for all analyses. Cox proportional hazards regression with Prentice weighting and robust standard errors was used to estimate multivariable-adjusted hazard ratios (HRs) for incident type 2 diabetes and their 95% confidence intervals (CI). The levels of blood metabolites at baseline were the exposure variable in the analysis. Age was used as the underlying time scale from recruitment to study exit (diagnosis of type 2 diabetes or censoring) of each participant. Covariates in the association analysis were age at baseline, sex, BMI, waist circumference, ethnicity, educational attainment, family history of type 2 diabetes, smoking status, alcohol consumption and level of self-reported physical activity.

For analyses stratified by quartiles, quartiles were defined by the distribution of metabolite levels in the sub-cohort and the HR of incident type 2 diabetes in each of the higher quartiles was estimated using the bottom quartile as reference category.

**5.** **Prospective association with incident type 2 diabetes of BCAA-pathway metabolites in the EPIC-Norfolk type 2 diabetes case-cohort study**

EPIC-Norfolk is a prospective cohort study of 25,639 individuals living in the Norfolk county in East Anglia (United Kingdom) [[17](#_ENREF_17)] and a constituent cohort of EPIC [[18](#_ENREF_18)]. The EPIC-Norfolk case-cohort study is the common subset of EPIC-Norfolk and InterAct, comprising 673 incident cases of type 2 diabetes and 830 non-cases. Details of genotyping in the EPIC-Norfolk case-cohort study are described above, in the paragraph about the EPIC-InterAct study. In the EPIC-Norfolk case-cohort study, BCAA levels were measured using an untargeted UPLC-MS/MS platform (DiscoveryHD4® platform - Metabolon Inc.) in citrated plasma samples [[19](#_ENREF_19)]. This is an updated version of the untargeted metabolomics platform employed by Shin and colleagues [[7](#_ENREF_7)]. In this study, we analysed not only the levels of the three BCAAs, but also those of an additional 15 metabolites related to BCAA-metabolism. Investigated metabolites were: isoleucine, leucine, valine, 3-methyl-2-oxovalerate, 3-methyl-2-oxobutyrate, 3-hydroxy-2-ethylpropionate, 4-methyl-2-oxopentanoate, 2-hydroxy-3-methylvalerate, ethylmalonate, 3-hydroxyisobutyrate, beta-hydroxyisovalerate, alpha-hydroxyisovalerate, alpha-hydroxyisocaproate, methylsuccinate, N-acetylvaline, propionylcarnitine, isovalerylcarnitine, and isobutyrylcarnitine.

Measurements were carried out using MS/MS instruments. For these measurements, instrument variability, determined by calculating the median relative standard deviation, was of 6%. Data Extraction and Compound Identification: Raw data was extracted, peak-identified and quality control-processed using Metabolon’s hardware and software. Compounds were identified by comparison to library entries of purified standards or recurrent unknown entities. Metabolon maintains a library, based upon authenticated standards, that contains the retention time/index (RI), mass to charge ratio (m/z), and chromatographic data (including MS/MS spectral data) of all molecules present in the library. Identifications were based on three criteria: retention index, accurate mass match to the library +/- 10 ppm, and the MS/MS forward and reverse scores between the experimental data and authentic standards. Metabolite Quantification and Data Normalization: Peaks were quantified using area-under-the-curve. A data normalization step was performed to correct variation resulting from instrument inter-day tuning differences. Essentially, each compound was corrected in run-day blocks by registering the medians to equal one (1.00) and normalizing each data point proportionately (termed the “block correction”).

*Statistical methods*

Before analysis, metabolite levels reported by Metabolon were log10-transformed, winsorised to 5 SDs and standardised to a mean of 0 and a standard deviation of 1. Cox proportional hazards regression with Prentice weighting and robust standard errors was used to estimate multivariable-adjusted hazard ratios (HRs) for incident type 2 diabetes and their 95% confidence intervals (CI). The levels of blood metabolites at baseline were the exposure variable in the analysis. Age was used as the underlying time scale from recruitment to study exit (diagnosis of type 2 diabetes or censoring) of each participant. Covariates in the association analysis were age at baseline, sex, BMI, waist circumference, ethnicity, educational attainment, family history of type 2 diabetes, smoking status, alcohol consumption and level of self-reported physical activity.

**6.** **Association of type 2 diabetes aetiological mechanisms with BCAA levels**

We investigated whether causal mechanisms in the pathophysiology of type 2 diabetes were associated with the levels of BCAAs. Investigated mechanisms were increased body mass index (BMI), increased insulin resistance (IR) and reduced early-phase insulin secretion (IS). We investigated the association with both observational (for BMI and IR) and (for the tree mechanisms) genetic analyses. Observational analyses were conducted in the Fenland study by testing the association of BMI and fasting insulin (exposures) with BCAA levels (outcome) in linear regression models adjusted for age and sex. Genetic analyses were conducted by testing the association of unweighted genetic scores for BMI, IR and IS (exposures) with BCAA levels (outcomes) in the Fenland, KORA and Twins UK studies. Validated genetic scores for BMI, IR and IS were taken from the literature [[20-22](#_ENREF_20)]. In the Fenland study, constituent SNPs were extracted and the allele dosage of the trait-raising allele was summed to compile the genetic scores. The association of genetic scores (exposure) with BCAA levels (outcomes) was tested by linear regression models adjusted for age, sex and genotyping chip. Results of the association between BMI, IR and IS SNPs with BCAA levels in a meta-analysis of KORA and Twins UK were extracted from the Metabolomics GWAS Server [[7](#_ENREF_7)]. These results were rescaled to 1 SD using the average of the reported SDs of a given metabolite in Twins UK and KORA [[7](#_ENREF_7)]. The association of multiple SNPs for a given score was pooled using the inverse-variance weighted method reported by Burgess et al. after assigning a weight of 1 to each SNP [[15](#_ENREF_15)]. We validated this approach in the Fenland study, where we had access to both results and individual level genotypes (see Table below). Because the lead SNPs at the *DDX19A* (rs75950518), *TRMT61A* (rs58101275) and *CBLN1* (rs1420601) are available in 1000 Genomes but not HapMap 2 (i.e. the reference panel used by Shin et al.) we sought for proxies in HapMap 2 using the SNAP software [[14](#_ENREF_14)] (r^2^ > 0.8). For *DDX19A,* we used rs12325419 as proxy of rs75950518 (r^2^ = 0.82). For *CBLN1*, we used rs1861569 as proxy of rs1420601 (r^2^ = 1). For *TRMT61A*, we could not find a suitable proxy. Results from the Fenland study and from the KORA and Twins UK studies were meta-analysed using fixed effect models.

**Table. Comparison of association results obtained using the methods by Burgess and colleagues [**[**15**](#_ENREF_15)**] or individual-level genotype data.**

| **Genetic score** | **Metabolite** | **Number of SNPs** | **Beta Burgess** | **Standard error Burgess** | **p-value Burgess** | **Beta genotypes** | **Standard error genotypes** | **p-value genotypes** |
| --- | --- | --- | --- | --- | --- | --- | --- | --- |
| BMI | Isoleucine | 32 | 0.006 | 0.0025 | 0.011 | 0.006 | 0.0025 | 0.012 |
| IR | Isoleucine | 10 | 0.015 | 0.0042 | 0.000 | 0.014 | 0.0043 | 0.001 |
| IS | Isoleucine | 20 | 0.000 | 0.0031 | 0.975 | 0.001 | 0.0033 | 0.775 |
| BMI | Leucine | 32 | 0.003 | 0.0027 | 0.236 | 0.004 | 0.0027 | 0.196 |
| IR | Leucine | 10 | 0.015 | 0.0045 | 0.001 | 0.014 | 0.0047 | 0.003 |
| IS | Leucine | 20 | -0.002 | 0.0034 | 0.648 | -0.001 | 0.0035 | 0.756 |
| BMI | Valine | 32 | 0.007 | 0.0027 | 0.007 | 0.007 | 0.0027 | 0.010 |
| IR | Valine | 10 | 0.010 | 0.0045 | 0.024 | 0.010 | 0.0047 | 0.039 |
| IS | Valine | 20 | 0.005 | 0.0034 | 0.183 | 0.005 | 0.0035 | 0.119 |

**7.** **Investigation of the association of BCAA-raising alleles with cardiometabolic traits**

We investigated the association of BCAA-raising alleles with BMI, waist-to-hip ratio adjusted for BMI, fasting insulin, fasting glucose, glucose at two hours in the course of an OGTT, triglycerides, HDL and LDL cholesterol in the result-level data of large-scale GWAS meta-analyses from consortia including the Genetic Investigation of ANthropometric Traits [[23](#_ENREF_23),[24](#_ENREF_24)], the Meta-Analyses of Glucose and Insulin-related traits Consortium [[25](#_ENREF_25),[26](#_ENREF_26)], and the Global Lipids Genetics Consortium [[27](#_ENREF_27)]. The sample size of each study is described in **S9 Table**. For the *TRMT61A* locus, which did not have a suitable proxy in HapMap, we used the Fenland study as data source. These investigations allowed an assessment of pleiotropy as well as of possible mediating mechanisms of the BCAA-raising alleles.

**8.** **Investigation of the association of BCAA-raising alleles with other metabolites**

In order to assess the specificity (i.e. absence of pleiotropy) of the genetic scores used in our Mendelian randomisation analysis, we tested their association with more than 500 metabolites in studies of the plasma metabolome. In the Fenland study, we tested the association of isoleucine, leucine and valine genetic scores with the levels of 175 metabolites from 6 biochemical classes. We applied a Bonferroni correction to adjudicate associations (i.e. 175 metabolites x 3 genetic scores [isoleucine, isoleucine with *GCKR* and leucine/valine] = 525 tests; P < 9.5 x 10^-5^). In KORA and TwinsUK studies, we tested the association of the BCAA-raising allele of rs1440581 at the *PPM1K* locus with the levels of 453 metabolites, applying a Bonferroni correction to adjudicate associations (i.e. 453 tests; P < 1.1 x 10^-4^).

**9.** **Analyses of metabolites specific to the BCAA pathway**

We tested the association of BCAA-raising alleles with up to 18 BCAA-pathway metabolites in the EPIC-Norfolk, KORA and Twins UK studies (total N = 8,693). In the EPIC-Norfolk study, we constructed unweighted genetic scores of isoleucine, leucine and valine using the lead SNPs from our meta-analysis of BCAA levels. Association analyses were performed using linear regression models adjusted for age, sex and diabetes status at follow-up. The unweighted genetic scores were the exposure variables and the metabolite levels were the outcome variables. Results of the association of BCAA-raising SNPs or their proxies with BCAA-pathway metabolites in KORA and Twins UK were extracted and rescaled to 1 SD using the average of the reported SDs of a given metabolite in Twins UK and KORA [[7](#_ENREF_7)]. The association of multiple SNPs for a given score was pooled using the inverse-variance weighted method reported by Burgess et al. [[15](#_ENREF_15)] after assigning a weight of 1 to each SNP [[15](#_ENREF_15)]. Where possible, results from the EPIC-Norfolk study and from the KORA and Twins UK studies were meta-analysed using fixed effect models. Because of the deeper coverage of metabolites in the updated Metabolon platform used in EPIC-Norfolk, some metabolites were available in that study only (i.e. 4-methyl-2-oxopentanoate, 2-hydroxy-3-methylvalerate, ethylmalonate, 3-hydroxyisobutyrate, beta-hydroxyisovalerate, alpha-hydroxyisovalerate, alpha-hydroxyisocaproate, methylsuccinate and N-acetylvaline). Association results were visualised using Metabolync®, a proprietary software of Metabolon. The position of BCAA-pathway metabolites relative to branched-chain alpha ketoacid dehydrogenase was manually annotated using annotations from the Kyoto Encyclopedia of Genes and Genomes (URL: http://www.genome.jp/kegg/) the Human Metabolome Database (URL: http://www.hmdb.ca/) and Metabolync® with the help of Metabolon staff.

**S1 Text References**

1. Illig T, Gieger C, Zhai G, Romisch-Margl W, Wang-Sattler R, et al. (2010) A genome-wide perspective of genetic variation in human metabolism. Nat Genet 42: 137-141.

2. Walsh BH, Broadhurst DI, Mandal R, Wishart DS, Boylan GB, et al. (2012) The metabolomic profile of umbilical cord blood in neonatal hypoxic ischaemic encephalopathy. PLoS One 7: e50520.

3. Johnson WE, Li C, Rabinovic A (2007) Adjusting batch effects in microarray expression data using empirical Bayes methods. Biostatistics 8: 118-127.

4. Howie BN, Donnelly P, Marchini J (2009) A flexible and accurate genotype imputation method for the next generation of genome-wide association studies. PLoS Genet 5: e1000529.

5. Marchini J, Howie B (2010) Genotype imputation for genome-wide association studies. Nat Rev Genet 11: 499-511.

6. Willer CJ, Li Y, Abecasis GR (2010) METAL: fast and efficient meta-analysis of genomewide association scans. Bioinformatics 26: 2190-2191.

7. Shin SY, Fauman EB, Petersen AK, Krumsiek J, Santos R, et al. (2014) An atlas of genetic influences on human blood metabolites. Nat Genet 46: 543-550.

8. Pruim RJ, Welch RP, Sanna S, Teslovich TM, Chines PS, et al. (2010) LocusZoom: regional visualization of genome-wide association scan results. Bioinformatics 26: 2336-2337.

9. InterAct C, Langenberg C, Sharp S, Forouhi NG, Franks PW, et al. (2011) Design and cohort description of the InterAct Project: an examination of the interaction of genetic and lifestyle factors on the incidence of type 2 diabetes in the EPIC Study. Diabetologia 54: 2272-2282.

10. Morris AP, Voight BF, Teslovich TM, Ferreira T, Segre AV, et al. (2012) Large-scale association analysis provides insights into the genetic architecture and pathophysiology of type 2 diabetes. Nat Genet 44: 981-990.

11. Kimber CH, Doney AS, Pearson ER, McCarthy MI, Hattersley AT, et al. (2007) TCF7L2 in the Go-DARTS study: evidence for a gene dose effect on both diabetes susceptibility and control of glucose levels. Diabetologia 50: 1186-1191.

12. Menni C, Fauman E, Erte I, Perry JR, Kastenmuller G, et al. (2013) Biomarkers for type 2 diabetes and impaired fasting glucose using a nontargeted metabolomics approach. Diabetes 62: 4270-4276.

13. Collins R (2012) What makes UK Biobank special? Lancet 379: 1173-1174.

14. Johnson AD, Handsaker RE, Pulit SL, Nizzari MM, O'Donnell CJ, et al. (2008) SNAP: a web-based tool for identification and annotation of proxy SNPs using HapMap. Bioinformatics 24: 2938-2939.

15. Burgess S, Butterworth A, Thompson SG (2013) Mendelian randomization analysis with multiple genetic variants using summarized data. Genet Epidemiol 37: 658-665.

16. Burgess S, Dudbridge F, Thompson SG (2015) Combining information on multiple instrumental variables in Mendelian randomization: comparison of allele score and summarized data methods. Stat Med.

17. Day N, Oakes S, Luben R, Khaw KT, Bingham S, et al. (1999) EPIC-Norfolk: study design and characteristics of the cohort. European Prospective Investigation of Cancer. Br J Cancer 80 Suppl 1: 95-103.

18. Riboli E, Hunt KJ, Slimani N, Ferrari P, Norat T, et al. (2002) European Prospective Investigation into Cancer and Nutrition (EPIC): study populations and data collection. Public Health Nutr 5: 1113-1124.

19. Evans AM, Bridgewater BR, Liu Q, Mitchell MW, Robinson RJ, et al. (2014) High resolution mass spectrometry improves data quantity and quality as compared to unit mass resolution mass spectrometry in high-throughput profiling metabolomics. Metabolomics: Open Access 4.

20. Scott RA, Fall T, Pasko D, Barker A, Sharp SJ, et al. (2014) Common genetic variants highlight the role of insulin resistance and body fat distribution in type 2 diabetes, independent of obesity. Diabetes 63: 4378-4387.

21. Nead KT, Sharp SJ, Thompson DJ, Painter JN, Savage DB, et al. (2015) Evidence of a Causal Association Between Insulinemia and Endometrial Cancer: A Mendelian Randomization Analysis. J Natl Cancer Inst 107.

22. Ostergaard SD, Mukherjee S, Sharp SJ, Proitsi P, Lotta LA, et al. (2015) Associations between Potentially Modifiable Risk Factors and Alzheimer Disease: A Mendelian Randomization Study. PLoS Med 12: e1001841; discussion e1001841.

23. Locke AE, Kahali B, Berndt SI, Justice AE, Pers TH, et al. (2015) Genetic studies of body mass index yield new insights for obesity biology. Nature 518: 197-206.

24. Shungin D, Winkler TW, Croteau-Chonka DC, Ferreira T, Locke AE, et al. (2015) New genetic loci link adipose and insulin biology to body fat distribution. Nature 518: 187-196.

25. Scott RA, Lagou V, Welch RP, Wheeler E, Montasser ME, et al. (2012) Large-scale association analyses identify new loci influencing glycemic traits and provide insight into the underlying biological pathways. Nat Genet 44: 991-1005.

26. Manning AK, Hivert MF, Scott RA, Grimsby JL, Bouatia-Naji N, et al. (2012) A genome-wide approach accounting for body mass index identifies genetic variants influencing fasting glycemic traits and insulin resistance. Nat Genet 44: 659-669.

27. Global Lipids Genetics C, Willer CJ, Schmidt EM, Sengupta S, Peloso GM, et al. (2013) Discovery and refinement of loci associated with lipid levels. Nat Genet 45: 1274-1283.
