# Supplementary material for: Genetic Predisposition to an Impaired Metabolism of the Branched-Chain Amino Acids and Risk of Type 2 Diabetes: A Mendelian Randomisation Analysis
Source: PLoS Med. 2016 Nov 29;13(11):e1002179. doi: 10.1371/journal.pmed.1002179 (PMC5127513; doi:10.1371/journal.pmed.1002179)
Supplement: S2 Text — (DOCX) [file pmed.1002179.s027.docx]

**S2 Text - Supplementary Results.**

*Section 1. The rs1260326 variant at the GCKR locus is not a suitable instrument for Mendelian randomisation analyses*

Mendelian randomisation assumes no pleiotropic effects of the genetic variants used as instrumental variables [[1](#_ENREF_1),[2](#_ENREF_2)]. Therefore, pleiotropic genetic variants are not a suitable instrument for Mendelian randomisation analyses. In this study, the rs1260326 variant at the *GCKR* locus was associated with the levels of isoleucine, but was excluded from the construction of the isoleucine genetic score, given that *GCKR* is a well-known pleiotropic locus [[3-7](#_ENREF_3)]. Accordingly, the *GCKR* rs1260326 variant was associated with body mass index (BMI), waist to hip ratio adjusted for BMI, fasting and two hour glucose, fasting insulin, HDL, LDL cholesterol and triglyceride levels (**S9 Table**). Adding the rs1260326 variant to the isoleucine genetic score isoleucine genetic score resulted in several pleiotropic associations with blood metabolites in the Fenland study and masked the association of the isoleucine genetic score with type 2 diabetes (**S9 Fig**). Overall, these data support the exclusion of rs1260326 from our analysis. It is important to note that, because polymorphisms at *GCKR* were not associated at genome-wide statistical significance with other BCAAs, the Mendelian randomisation analyses for those are not affected by *GCKR* variants-derived pleiotropy.

Besides the lack of utility in Mendelian randomisation analysis, it is possible that some of the pleiotropic associations at the *GCKR* locus are explained by a metabolic switch in the liver that drives metabolism toward triglyceride (and perhaps BCAAs) rather than glucose production as suggested by work from Beer et al. [[8](#_ENREF_8)] and Orho-Melander and colleagues [[9](#_ENREF_9)].

*Section 2. Systematic review of the observational association between baseline BCAA levels and incident type 2 diabetes*

A workflow of the systematic review of the literature about the prospective association between BCAA levels and incident type 2 diabetes is presented in **S4 Fig**. We extracted data on the relative risk of type 2 diabetes per 1 SD difference in isoleucine, leucine or valine from five prospective studies. Results were meta-analysed with the unpublished results of the EPIC-Norfolk case-cohort study. The characteristics of the studies included in the meta-analysis are reported in **S3 Table**. The total sample size of these analyses was of 1,992 cases of incident type 2 diabetes and 4,319 controls. **S5 Fig** reports the results of the meta-analysis. All studies reported a relative increase in type 2 diabetes risk for individuals with higher BCAA levels. The I-squared statistic was consistent with no/low heterogeneity in the relative risk estimates. **S7 Table** reports the results of the meta-analysis of quartile associations for isoleucine in 1,025 incident cases and 1,182 controls.

**S2 Text – References.**

1. Burgess S, Butterworth A, Thompson SG (2013) Mendelian randomization analysis with multiple genetic variants using summarized data. Genet Epidemiol 37: 658-665.

2. Smith GD, Ebrahim S (2003) 'Mendelian randomization': can genetic epidemiology contribute to understanding environmental determinants of disease? Int J Epidemiol 32: 1-22.

3. Scott RA, Lagou V, Welch RP, Wheeler E, Montasser ME, et al. (2012) Large-scale association analyses identify new loci influencing glycemic traits and provide insight into the underlying biological pathways. Nat Genet 44: 991-1005.

4. Manning AK, Hivert MF, Scott RA, Grimsby JL, Bouatia-Naji N, et al. (2012) A genome-wide approach accounting for body mass index identifies genetic variants influencing fasting glycemic traits and insulin resistance. Nat Genet 44: 659-669.

5. Locke AE, Kahali B, Berndt SI, Justice AE, Pers TH, et al. (2015) Genetic studies of body mass index yield new insights for obesity biology. Nature 518: 197-206.

6. Shungin D, Winkler TW, Croteau-Chonka DC, Ferreira T, Locke AE, et al. (2015) New genetic loci link adipose and insulin biology to body fat distribution. Nature 518: 187-196.

7. Global Lipids Genetics C, Willer CJ, Schmidt EM, Sengupta S, Peloso GM, et al. (2013) Discovery and refinement of loci associated with lipid levels. Nat Genet 45: 1274-1283.

8. Beer NL, Tribble ND, McCulloch LJ, Roos C, Johnson PR, et al. (2009) The P446L variant in GCKR associated with fasting plasma glucose and triglyceride levels exerts its effect through increased glucokinase activity in liver. Hum Mol Genet 18: 4081-4088.

9. Orho-Melander M, Melander O, Guiducci C, Perez-Martinez P, Corella D, et al. (2008) Common missense variant in the glucokinase regulatory protein gene is associated with increased plasma triglyceride and C-reactive protein but lower fasting glucose concentrations. Diabetes 57: 3112-3121.
